# Exploring the Origins and Evolution of Oxygenic and Anoxygenic Photosynthesis in Deeply Branched *Cyanobacteriota*

**DOI:** 10.1093/molbev/msae151

**Published:** 2024-07-23

**Authors:** Sha Tan, Lan Liu, Jian-Yu Jiao, Meng-Meng Li, Chao-Jian Hu, Ai-Ping Lv, Yan-Ling Qi, Yu-Xian Li, Yang-Zhi Rao, Yan-Ni Qu, Hong-Chen Jiang, Rochelle M Soo, Paul N Evans, Zheng-Shuang Hua, Wen-Jun Li

**Affiliations:** State Key Laboratory of Biocontrol, School of Life Sciences, Sun Yat-Sen University, Guangzhou 510275, PR China; Guangdong Provincial Key Laboratory of Plant Stress Biology, Sun Yat-Sen University, Guangzhou 510275, PR China; Southern Marine Science and Engineering Guangdong Laboratory (Zhuhai), Sun Yat-Sen University, Guangzhou 510275, PR China; State Key Laboratory of Biocontrol, School of Life Sciences, Sun Yat-Sen University, Guangzhou 510275, PR China; Guangdong Provincial Key Laboratory of Plant Stress Biology, Sun Yat-Sen University, Guangzhou 510275, PR China; Southern Marine Science and Engineering Guangdong Laboratory (Zhuhai), Sun Yat-Sen University, Guangzhou 510275, PR China; State Key Laboratory of Biocontrol, School of Life Sciences, Sun Yat-Sen University, Guangzhou 510275, PR China; Guangdong Provincial Key Laboratory of Plant Stress Biology, Sun Yat-Sen University, Guangzhou 510275, PR China; Southern Marine Science and Engineering Guangdong Laboratory (Zhuhai), Sun Yat-Sen University, Guangzhou 510275, PR China; State Key Laboratory of Biocontrol, School of Life Sciences, Sun Yat-Sen University, Guangzhou 510275, PR China; Guangdong Provincial Key Laboratory of Plant Stress Biology, Sun Yat-Sen University, Guangzhou 510275, PR China; Southern Marine Science and Engineering Guangdong Laboratory (Zhuhai), Sun Yat-Sen University, Guangzhou 510275, PR China; State Key Laboratory of Biocontrol, School of Life Sciences, Sun Yat-Sen University, Guangzhou 510275, PR China; Guangdong Provincial Key Laboratory of Plant Stress Biology, Sun Yat-Sen University, Guangzhou 510275, PR China; Southern Marine Science and Engineering Guangdong Laboratory (Zhuhai), Sun Yat-Sen University, Guangzhou 510275, PR China; State Key Laboratory of Biocontrol, School of Life Sciences, Sun Yat-Sen University, Guangzhou 510275, PR China; Guangdong Provincial Key Laboratory of Plant Stress Biology, Sun Yat-Sen University, Guangzhou 510275, PR China; Southern Marine Science and Engineering Guangdong Laboratory (Zhuhai), Sun Yat-Sen University, Guangzhou 510275, PR China; Chinese Academy of Sciences Key Laboratory of Urban Pollutant Conversion, Department of Environmental Science and Engineering, University of Science and Technology of China, Hefei 230026, PR China; Chinese Academy of Sciences Key Laboratory of Urban Pollutant Conversion, Department of Environmental Science and Engineering, University of Science and Technology of China, Hefei 230026, PR China; Chinese Academy of Sciences Key Laboratory of Urban Pollutant Conversion, Department of Environmental Science and Engineering, University of Science and Technology of China, Hefei 230026, PR China; Chinese Academy of Sciences Key Laboratory of Urban Pollutant Conversion, Department of Environmental Science and Engineering, University of Science and Technology of China, Hefei 230026, PR China; State Key Laboratory of Biogeology and Environmental Geology, China University of Geosciences, Wuhan 430074, PR China; The University of Queensland, School of Chemistry and Molecular Biosciences, Australian Centre for Ecogenomics, St Lucia, QLD 4072, Australia; The University of Queensland, School of Chemistry and Molecular Biosciences, Australian Centre for Ecogenomics, St Lucia, QLD 4072, Australia; Chinese Academy of Sciences Key Laboratory of Urban Pollutant Conversion, Department of Environmental Science and Engineering, University of Science and Technology of China, Hefei 230026, PR China; State Key Laboratory of Biocontrol, School of Life Sciences, Sun Yat-Sen University, Guangzhou 510275, PR China; Guangdong Provincial Key Laboratory of Plant Stress Biology, Sun Yat-Sen University, Guangzhou 510275, PR China; Southern Marine Science and Engineering Guangdong Laboratory (Zhuhai), Sun Yat-Sen University, Guangzhou 510275, PR China; State Key Laboratory of Desert and Oasis Ecology, Key Laboratory of Ecological Safety and Sustainable Development in Arid Lands, Xinjiang Institute of Ecology and Geography, Chinese Academy of Sciences, Urumqi 830011, PR China

**Keywords:** *Cyanobacteriota*, oxygenic photosynthesis, anoxygenic photosynthesis, thylakoid membrane, sulfide oxidation

## Abstract

*Cyanobacteriota*, the sole prokaryotes capable of oxygenic photosynthesis (OxyP), occupy a unique and pivotal role in Earth's history. While the notion that OxyP may have originated from *Cyanobacteriota* is widely accepted, its early evolution remains elusive. Here, by using both metagenomics and metatranscriptomics, we explore 36 metagenome-assembled genomes from hot spring ecosystems, belonging to two deep-branching cyanobacterial orders: *Thermostichales* and *Gloeomargaritales*. Functional investigation reveals that *Thermostichales* encode the crucial thylakoid membrane biogenesis protein, vesicle-inducing protein in plastids 1 (Vipp1). Based on the phylogenetic results, we infer that the evolution of the thylakoid membrane predates the divergence of *Thermostichales* from other cyanobacterial groups and that *Thermostichales* may be the most ancient lineage known to date to have inherited this feature from their common ancestor. Apart from OxyP, both lineages are potentially capable of sulfide-driven AnoxyP by linking sulfide oxidation to the photosynthetic electron transport chain. Unexpectedly, this AnoxyP capacity appears to be an acquired feature, as the key gene *sqr* was horizontally transferred from later-evolved cyanobacterial lineages. The presence of two D1 protein variants in *Thermostichales* suggests the functional flexibility of photosystems, ensuring their survival in fluctuating redox environments. Furthermore, all MAGs feature streamlined phycobilisomes with a preference for capturing longer-wavelength light, implying a unique evolutionary trajectory. Collectively, these results reveal the photosynthetic flexibility in these early-diverging cyanobacterial lineages, shedding new light on the early evolution of *Cyanobacteriota* and their photosynthetic processes.

## Introduction

Oxygenic photosynthesis (OxyP) is undoubtedly one of the most significant biological developments in Earth's history, transforming the Earth's atmosphere and paving the way for the subsequent emergence of complex life (Hohmann-Marriott and Blankenship 2011). Among the known prokaryotes, *Cyanobacteriota* (formerly called *Cyanobacteria*; Oren et al. 2022) is the only phylum capable of OxyP, and phototrophy in eukaryotes is widely believed to be obtained through the cyanobacterial endosymbiont (Hohmann-Marriott and Blankenship 2011; Fischer et al. 2016). Yet, the emergence and evolution of photosynthetic capabilities in the common ancestor of *Cyanobacteriota* remain cryptic. Several studies have proposed that OxyP in *Cyanobacteriota* evolved from anoxygenic photosynthesis (AnoxyP; Mulkidjanian et al. 2006; Martin et al. 2018). Alternatively, others have argued that photosystems were capable of H_2_O oxidation from the beginning, implying that the most recent common ancestor of *Cyanobacteriota* already evolved the ability to perform OxyP (Blank and Sánchez-Baracaldo 2010; Cardona and Rutherford 2019). Furthermore, based on the fact that all members of the two basal cyanobacterial classes, *Vampirovibrionia* and *Sericytochromatia*, have been found to completely lack genes for photosynthesis, some researchers have proposed that cyanobacterial ancestors were non-photosynthetic and that the acquisition of photosynthetic genes was relatively late in the evolution of *Cyanobacteriota* (Di Rienzi et al. 2013; Soo et al. 2017). OxyP relies on two reaction centers (RCs; type I and type II RCs; or photosystem I [PI] and photosystem II [PII]) to oxidize water and release oxygen as a by-product. In contrast, AnoxyP relies on only one RC (either type I or type II) to oxidize alternative electron donors, such as reduced sulfur compounds, hydrogen, or Fe^2+^, and does not produce oxygen (Mulkidjanian et al. 2006; Johnston et al. 2009). To date, all known extant *Cyanobacteriota* capable of performing AnoxyP were derived from sulfide-containing environments, where they can utilize sulfide as an electron donor for photoautotrophic growth (Cohen et al. 1975; Garcia-Pichel and Castenholz 1990; Klatt et al. 2015; Hamilton et al. 2018).

To understand the evolutionary processes of photosynthesis within *Cyanobacteriota*, it is crucial to explore the deeply branched cyanobacterial lineages. *Gloeobacterales* is the earliest diverging lineage among extant photosynthetic *Cyanobacteriota*, and a number of studies have revealed its unique characteristics, such as the lack of thylakoid, simplified photosystems, and unusual phycobilisome morphology (Saw et al. 2013; Grettenberger et al. 2020; Rahmatpour et al. 2021). Adjacent to *Gloeobacterales* is the subclade G lineage, which consists of *Synechococcus* sp. JA-3-3Ab, *Synechococcus* sp. JA-2-3B’a, and *Synechococcus* PCC 7336 strains (Shih et al. 2013). This grouping has subsequently been classified as an order of the phylum *Cyanobacteriota* in the Genome Taxonomy Database (GTDB) r214, named *Thermostichales*. These isolates, *Synechococcus* sp. JA-3-3Ab and JA-2-3B'a, are ecologically distinct as they predominate at different temperatures in the microbial mats of Octopus spring in Yellowstone National Park (Allewalt et al. 2006; Bhaya et al. 2007). In addition, *Gloeomargaritales* is another important deep-branching lineage, as *Gloeomargarita lithophora* has been reported as the closest known relative of chloroplasts, which provides new clues to the emergence of photosynthetic eukaryotes (Ponce-Toledo et al. 2017; Sánchez-Baracaldo et al. 2017).

Despite the aforementioned glimpses into the diversity of basal cyanobacterial lineages, their metabolic characteristics and the evolution of photosynthesis remain unclear due to the lack of genome representatives. To address this gap, we analyzed 36 cyanobacterial metagenome-assembled genomes (MAGs) from two deep-branching lineages (*Thermostichales* and *Gloeomargaritales*) obtained from 22 hot spring sediments in Yunnan and Tibet, China. Our findings reveal that *Thermostichales* possesses ancient key genes for the biogenesis of the thylakoid membrane, and it is highly probable that the evolution of this gene may predate the divergence of *Thermostichales*. Genomic analyses indicate the potential photosynthetic versatility in members of these two lineages, equipped with both OxyP and sulfide-driven AnoxyP. The evolution of AnoxyP in these genomes is attributed to horizontal acquisition of the *sqr* gene from other *Cyanobacteriota*. These findings significantly expand our knowledge of deeply branched *Cyanobacteriota* and provide valuable insights into the evolution of photosynthesis.

## Results

### *Thermostichales* and *Gloeomargaritales* Genomes Recovered from Metagenomes

**Fig. 1.**
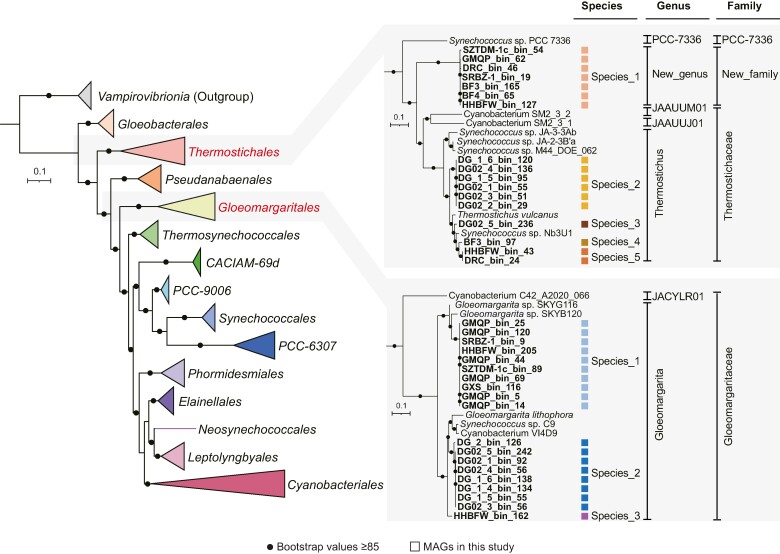
Phylogenetic analysis of the MAGs. The maximum-likelihood tree was constructed based on the concatenation of 31 marker genes. References from the class *Vampirovibrionia* were treated as outgroups. Branches are colored according to the GTDB r214 order-level classification. Nodes with bootstrap value ≥ 85 are indicated as solid circle. Thirty-six MAGs from this study are labeled bold and marked by colored square in enlarged phylogenetic tree.

A total of 36 MAGs belonging to the phylum *Cyanobacteriota* were obtained from 22 sediment metagenomes that were sequenced from 8 hot springs located in AngDa county of Tibet and Tengchong county of Yunnan, China (supplementary table S1, Supplementary Material online). The genome sizes of these reconstructed MAGs range from 1.3 to 3.5 Mbp. Genome quality evaluated by CheckM (Parks et al. 2015) reveals that most MAGs are of high quality, with genome completeness ranging from 61.2% to 99.1%, half of which exceeded 90% completeness, with nearly no contamination (0% to 0.43%; supplementary table S2, Supplementary Material online). The topologies of two maximum-likelihood trees constructed based on 31 conserved proteins (Fig. 1) and 120 bacterial marker proteins (supplementary fig. S1, Supplementary Material online) are consistent with each other, placing 19 and 17 of the cyanobacterial MAGs into *Thermostichales* and *Gloeomargaritales*, respectively. The average nucleotide identity (ANI) analysis identifies eight species using a 95% ANI cutoff (supplementary table S3, Supplementary Material online). MAGs from *Gloeoemargaritales* share 75.6% to 84.0% average amino acid identity (AAI; an acknowledged genus-level threshold range between 65% and 72% AAI; Konstantinidis and Tiedje 2007) and 94.2% to 98.4% of 16S rRNA gene sequence identities with *Gloeomargarita lithophora* Alchichica-D10, suggesting they might belong to the same genus (supplementary tables S4 and S5, Supplementary Material online). MAGs from *Thermostichales* can be divided into two distinct families. MAGs of *Thermostichales* species1 share 57.4% to 64.1% AAI with eight *Thermostichales* reference genomes, which were lower than the genus-level threshold (supplementary table S4, Supplementary Material online). Moreover, combined with their average relative evolutionary divergence (RED) values (0.70 ± 0.003) and monophyly with high bootstrap support, MAGs belonging to *Thermostichales*_species1 could be assigned to a novel family (Fig. 1). AAI values between the remaining MAGs of *Thermostichales* and *Thermostichus vulcanus* are 86.2% to 90.9%, suggesting these MAGs can be assigned to the genus *Thermostichus* within the family *Thermostichaceae* (supplementary table S4, Supplementary Material online).

### *Thermostichales* Contain Ancient Enzyme for Thylakoid Biogenesis

**Fig. 2.**
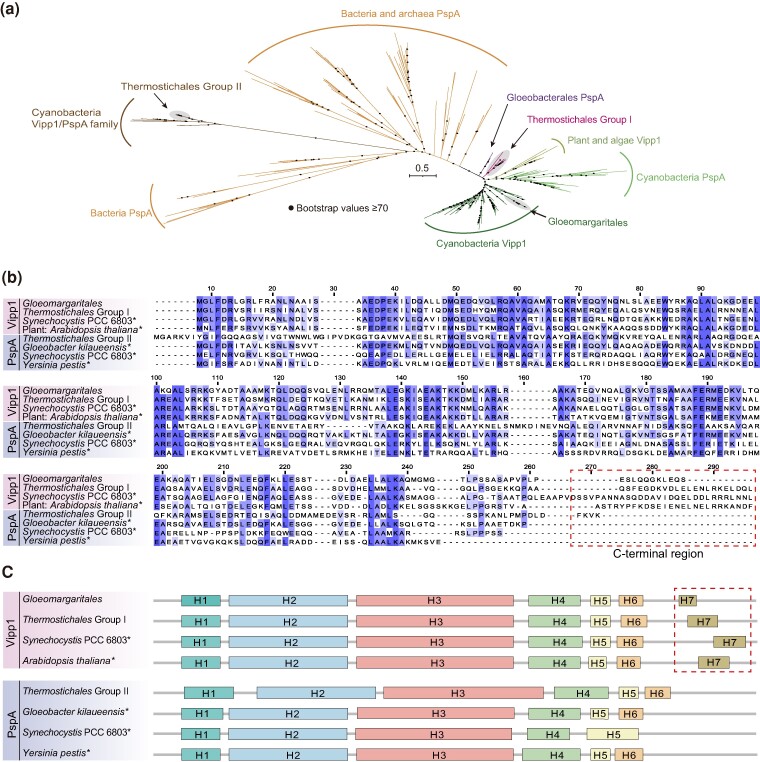
Analysis of Vipp1/PspA family proteins from the MAGs. a) Unrooted maximum-likelihood tree of Vipp1 and PspA proteins from MAGs in this study and various organisms was constructed by IQ-TREE with 1,000 ultrafast bootstrapping iterations. The Vipp1 and PspA cluster from different phylogenetic groups were labeled. Branches circled by ellipses represent sequences from the MAGs. b) Sequence alignment of selected Vipp1 and PspA proteins from each cluster identified in a). Amino acid residues sharing more than 50% identidy are colored in columns. The C-terminal extension of sequences are marked by dotted rectangle. Confirmed reference sequences of Vipp1 and PspA are marked with asterisks. c) Secondary structures of selected Vipp1 and PspA proteins. α-helices are labeled H1–H7, and the α-helical extension (H7) at the C-terminus of Vipp1 proteins are marked by dotted rectangle.

Vipp1 plays an essential role in the biosynthesis of thylakoid membranes, which are photosynthetically active membranes found in *Cyanobacteriota* and chloroplasts (Heidrich et al. 2017). It was assumed that Vipp1 may have evolved from the homologous phage shock protein PspA through gene duplication (Westphal et al. 2001). PspA is highly homologous to Vipp1 and is primarily involved in bacterial stress responses and membrane repair (Vothknecht et al. 2012). Nevertheless, Vipp1 features a C-terminal extension that forms an additional α-helix, distinguishing it from PspA (Vothknecht et al. 2012). This C-terminal tail is presumed to regulate the flexibility of Vipp1 particle association and dissociation, which is crucial for maintaining membrane integrity and modulating membrane fusion (Zhang et al. 2016; Hennig et al. 2017). A total of 45 Vipp1/PspA proteins were identified from MAGs in this study, and most MAGs in *Thermostichales* contained two copies. Previous study has also reported the presence of genes encoding Vipp1/PspA homologs in *Synechococcus* sp. PCC 7336, JA-3-3Ab, and JA-2-3B’ (Mareš et al. 2019). Phylogenetic analysis revealed that *Gloeomargaritales* Vipp1/PspA placed within the cyanobacterial Vipp1 clade, while Vipp1/PspA from *Thermostichales* formed two different lineages, *Thermostichales* group I and *Thermostichales* group II (Fig. 2a). Noticeably, sequences of *Thermostichales* group I branch outside of the cyanobacterial and plant Vipp1/PspA clades and adjacent to the proteins of *Gloeobacterales*, the deepest-branching group of all known oxygenic photosynthetic *Cyanobacteriota*. *Gloeobacterales* is the only known cyanobacterial group that lacks thylakoids, and thus, its photosynthetic machinery is located in the plasma membrane instead of the thylakoid membranes (Raven and Sánchez-Baracaldo 2021). Despite the presence of Vipp1/PspA family proteins in *Gloeobacterales*, they all lack the C-terminal extension that is unique to Vipp1, leading to their annotation as PspA homologs (Fig. 2b and c). This observation further supports the thylakoid-specific function of Vipp1. Additionally, the Vipp1/PspA proteins from *Thermostichales* group II and several cyanobacterial species form separate branches, diverging from the cyanobacterial Vipp1 and PspA clade and clustering with the bacterial PspA (Fig. 2a). By looking into the sequence alignment of these proteins, we infer that those proteins from *Thermostichales* group I and *Gloeomargaritales* were Vipp1 due to the detection of the C-terminal extension in their amino acid sequences (Fig. 2b). The secondary and crystal structure predictions of these proteins consolidated this inference since the extra region encoding an α-helix shared a similar structure to experimentally verified Vipp1 in *Arabidopsis thaliana* (Kroll et al. 2001) and *Synechocystis* sp. PCC 6803 (Westphal et al. 2001) (Fig. 2c and supplementary fig. S2, Supplementary Material online). Given the basal position of *Thermostichales* in both the phylogenomic tree and the Vipp1 protein tree (Figs. 1 and 2a), we reason that the *vipp1* gene evolved before the divergence of *Thermostichales*, and this lineage is probably the earliest cyanobacterial group known to date, which have acquired this capacity from their common ancestor. In contrast, all Vipp1/PspA proteins from the *Thermostichales* group II lack the α-helix at the C-terminus; therefore, these proteins are likely to be PspA. Furthermore, genes required for the synthesis of four major lipids in thylakoid membranes were found in the MAGs of *Thermostichales* and *Gloeomargaritales* but not in thylakoid-lacking *Gloeobacterales* species (supplementary table S6, Supplementary Material online). Taken together, both lineages in the present study harbor the Vipp1 protein, and the origin of thylakoid membrane predates the divergence of *Thermostichales*. The development of this photosynthetic membrane structure also profoundly affected the further evolution of photosynthesis in *Cyanobacteriota*.

### Both *Thermostichales* and *Gloeomargaritales* Have Streamlined Phycobilisomes

**Fig. 3.**
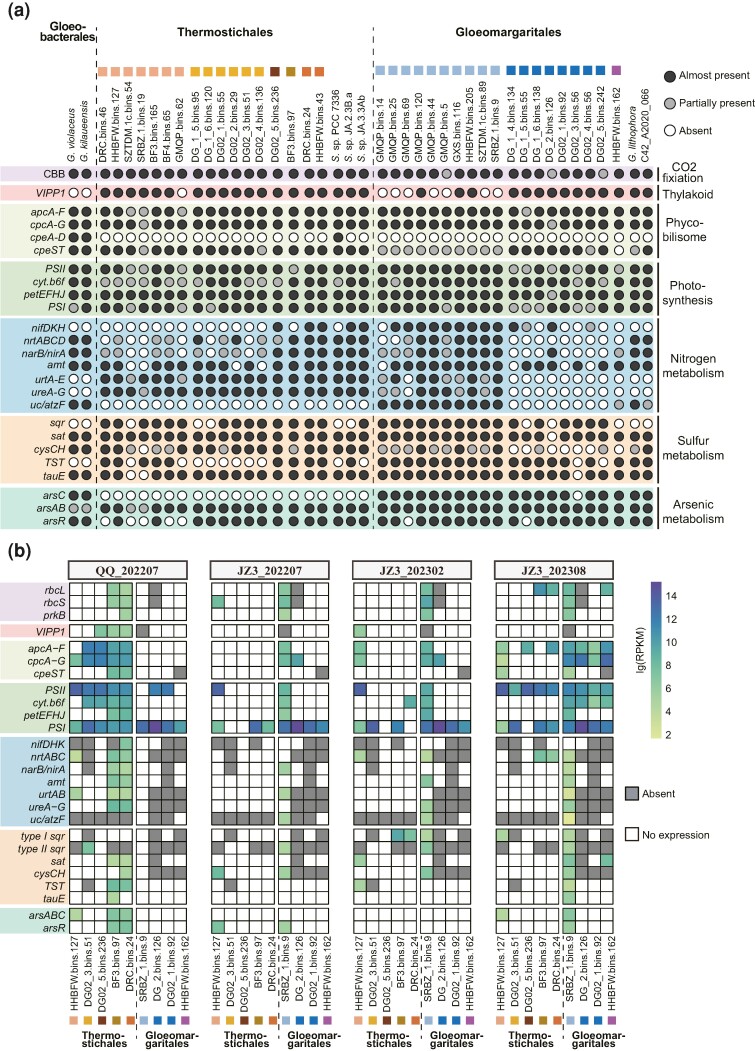
The distribution and in situ activities of selected metabolic features in MAGs of *Thermostichales* and *Gloeomargaritales*. a) Black and white circles indicate gene/pathway presence and absence, respectively. The gray circle represents a partial pathway (related genes exist less than 70%). A complete list of genes is provided in supplementary table S7, Supplementary Material online. b) Expression profiles of genes from nine representative MAGs in four metatranscriptomes. Gray represents a gene that is absent, and white indicates that gene is not expressed. The in situ activity of each gene is detailed in supplementary fig. S5 and table S8, Supplementary Material online.

Phycobilisomes are aggregates of phycobiliproteins attached to the cytoplasmic surface of the thylakoid membrane and serve as the major accessory light-harvesting complex in *Cyanobacteriota*. Phycobiliproteins can be classified into four types based on their spectral properties and pigment compositions: allophycocyanin (APC; λmax = 650 to 660 nm), phycocyanin (PC; λmax = 610 to 625 nm), phycoerythrin (PE; λmax = 490 to 570 nm), and phycoerythrocyanin (PEC; λmax = 560 to 600 nm; Pagels et al. 2019). All MAGs from this study lack virtually all genes encoding PE or PEC, illustrating that the phycobilisomes in these deep-branching microbes do not optimally capture short-wavelength light, such as green (492 to 577 nm) and yellow (577 to 597 nm) lights (Fig. 3a). The only detected genes, *cpeS* and *cpeT*, involved in PE can be used to catalyze the covalent attachment of chromophore to various phycobiliproteins, such as PC (Zhao et al. 2007; Zhang et al. 2014). However, complete PE-associated genes were found in *Synechococcus* sp. PCC 7336 and species of *Gloeobacterales*. It is reported that PE may have evolved through the complex processes of gene replication, horizontal gene transfer, and gene loss (Six et al. 2007; Tanabe and Yamaguchi 2018), which have contributed to the patchy distribution of PE in these basal cyanobacterial lineages. Moreover, the loss of PE in some eukaryotic algae and cyanobacteria is believed to serve a dual purpose: assisting them in adapting to high-light conditions and reallocating resources that were initially dedicated to the formation of complete phycobilisomes (Ritz et al. 2000; Chrismas et al. 2018). Thus, we can hypothesize that the absence of PE and PEC in these MAGs may represent a potential adaptive strategy that could reduce the risk of photoinhibition by limiting the spectral absorption range and conserve material and energy resources. Collectively, the phycobilisomes of MAGs in the present study lack PE and PEC and thus may screen short-wavelength light. Instead, they likely exploit low-light microenvironments, optimally capturing long-wavelength, such as red (620 to 700 nm) and orange (597 to 620 nm) lights through APC and PC to harvest energy.

### Some *Thermostichales* Genomes Lack a Few Photosynthetic Auxiliary Genes

The light energy absorbed by phycobilisomes is transferred to PSII and PSI, two vital components of OxyP in the thylakoid membranes. Aside from MAGs with low quality, virtually all MAGs in *Gloeomargaritales* and *Thermostichales* harbor key subunits of PSI and PSII complexes (Fig. 3a). However, photosystem auxiliary genes, including *psaI*, *psaM*, *psbI*, and *psbM*, are not detected in several species of *Thermostichales* (supplementary table S7, Supplementary Material online). PsaI and PsaM subunits are involved in linking PSI monomers into trimers (Sener et al. 2004; Xu et al. 2021), and similarly, PsbI and PsbM subunits are responsible for the formation and stability of PSII dimers (Kawakami et al. 2011). Furthermore, the cytochrome *b_6_f* (Cyt *b_6_f*) complex is a crucial component of the photosynthetic electron transport chain, linking the electron transport between PSII and PSI. The Cyt *b_6_f* in *Cyanobacteriota* is typically composed of eight subunits. However, all of the MAGs in this study lack the *petL* gene, and a substantial number of *Thermostichales* MAGs are also devoid of either *petM* or *petN* (supplementary table S7, Supplementary Material online). Both PetL and PetM are considered non-essential subunits, with PetL is involved in the stabilization and formation of the dimeric Cyt *b_6_f* complex (Schwenkert et al. 2007), while PetM has regulatory roles (Schneider et al. 2001). PetN appears to be crucial for Cyt *b_6_f* stability and photoautotrophic growth in plants, but its role in *Cyanobacteriota* remain unclear, as the *petN* gene cannot be completely knocked out (Schneider et al. 2007; Schwenkert et al. 2007). Although none of these missing auxiliary genes are essential for OxyP (Rahmatpour et al. 2021), the streamlined photosynthetic systems in *Thermostichales* may exhibit somewhat diminished stability and activity. However, we still cannot rule out the possibility that alternative regulation genes are employed by the *Thermostichales* MAGs. Metatranscriptomic analysis revealed that *Thermostichales* and *Gloeomargaritales* account for only a few transcripts in the community photosynthetic RC gene set (*psaA*, *psaB*, *psbA*, *psbD*, *pscA*, *pshA*, *pufL*, and *pufM*; supplementary fig. S3, Supplementary Material online). Nonetheless, genes associated with photosystems and phycobilisomes were expressed at a high level by representative *Thermostichales* and *Gloeomargaritales* MAGs in four metatranscriptomes, which suggests photosynthesis may be the key metabolism for harvesting light energy by these microbes in hot spring ecosystems (Fig. 3b; supplementary fig. S4 and supplementary table S8, Supplementary Material online). Collectively, MAGs from both lineages exhibit the capability for OxyP, with certain *Thermostichales* MAGs displaying a more simplified set of photosystem subunits.

### Sulfide Oxidation Enables Possible Phototrophy and Chemotrophy in *Thermostichales* and *Gloeomargaritales*

**Fig. 4.**
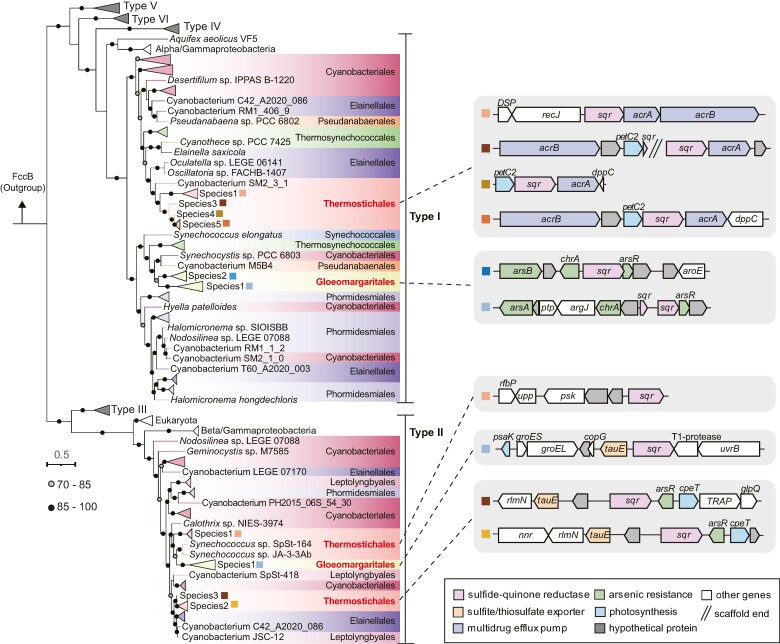
Phylogenetic tree of SQR and the organization of *sqr*-related gene cluster in the MAGs. The tree was inferred using IQ-TREE with 1,000 ultrafast bootstrapping iterations and only bootstrap values higher than 70% are shown. Sequences of flavocytochrome c–sulfide dehydrogenase (FccB) were treated as outgroups. Cyanobacterial branches are colored according to the order-level classification. MAGs assembled from the present study are marked by colored square. The right panel is the schematic diagram of *sqr* gene operon of MAGs from this study.

Most *Gloeomargaritales* and *Thermostichales* MAGs contain one or two copies of sulfide–quinone oxidoreductases (SQR), which is a membrane-associated protein that oxidizes sulfide to zero-valent sulfur (polysulfide). In addition to its role in sulfide detoxification, SQR can be involved in cyanobacterial AnoxyP, which oxidizes sulfide and transfers electrons to PSI, bypassing PSII (Klatt et al. 2015). Sequences from *Gloeomargaritales* and *Thermostichales* MAGs belong to type I and II SQR (Fig. 4). Surprisingly, phylogenetic analysis revealed that the *sqr* genes in these two deep-branching lineages were horizontally transferred from subsequently diversified cyanobacterial clades, specifically those within the *Cyanobacteriales* and *Elainellales* (Fig. 4). The amino acid sequences of type I SQR from two lineages show 48% to 66% and 54% to 59% identities to the functionally well-characterized SQR from *Geitlerinema* sp. PCC 9228 and *Aphanothece halophytica*, which have been validated to be involved in sulfidotrophic AnoxyP in these cyanobacterial isolates (Arieli et al. 1994; Bronstein et al. 2000). The type II SQR sequences from MAGs showed 51% to 60% identities to those of *Chloroflexus* species, which have been shown to be capable of using sulfide as an electron donor for autotrophic growth (Thiel et al. 2014; Kanno et al. 2019). Moreover, metatranscriptomic data confirmed the expression of *sqr* genes in some representative MAGs (Fig. 3b and supplementary fig. S5, Supplementary Material online).

Also, gene cluster surveys revealed that arsenic-related *sqr* clusters were detected in *Gloeomargaritales* MAGs, with the presence of the arsenite transcriptional repressor (ArsR), arsenite transporting ATPase (ArsA), arsenical pump membrane protein (ArsB), and chromatin/arsenite uptake transporter (ChrA) adjacent to type I *sqr* genes (Fig. 4). A similar gene cluster occurs in *Synechocystis* sp. PCC6803, which has the ability to utilize sulfide as an electron donor for AnoxyP under anoxic conditions (Nagy et al. 2014). Considering the toxicity of sulfide and arsenite, the arsenic-related *sqr* cluster may also contribute to the detoxification and alleviate the inhibitory effect of sulfide on PSII since both substrates can be commonly detected in hot spring ecosystems formed by volcanic eruptions or other geological activities. In MAGs of *Thermostichales* species3, species4, and species5, the type I *sqr* gene is flanked by *petC2*, which encodes the Rieske protein of cytochrome *b_6_f* complex (Fig. 4). Previous studies show that PetC2 can replace the major Rieske isoform PetC1 to participate in photosynthesis and respiration, and gene expressions of *petC2* adjacent to *sqr* were upregulated in several cyanobacterial species under low oxygen concentrations (Summerfield et al. 2008; Schultze et al. 2009). This suggests that *petC2* and the adjacent *sqr* gene in MAGs from this study may also be involved in respiratory and photosynthetic electron transport under hypoxic conditions. Akin to the type I *sqr* clusters in *Gloeomargaritales*, *Thermostichales* MAGs harbored the *arsR* gene upstream of type II *sqr*, demonstrating similar transcriptional regulation to resist arsenic stress (Fig. 4). Despite the lack of *chrA*, the sulfite and/or thiosulfate exporter *tauE* is found adjacent to most type II *sqr* genes. It is worth noting the presence of thiosulfate sulfotransferase (TST)/rhodanese in these MAGs (Fig. 3a). This enzyme is crucial in the mitochondrial sulfide oxidation pathway, catalyzing the conversion of sulfite and persulfide to thiosulfate (Hildebrandt and Grieshaber 2008; Libiad et al. 2014). However, this pathway is incomplete due to the lack of persulfide dioxygenase, which is necessary for sulfite production. Instead, we observed the presence of genes related to assimilatory sulfate reduction (*sat*, *cysC*, and *cysH*), which could replenish sulfite (Fig. 3a; supplementary fig. S12, Supplementary Material online). Therefore, the incomplete mitochondrial sulfide oxidation pathway, coupled with assimilatory sulfate reduction, may serve as an alternative strategy for sulfide detoxification. The nontoxic end-product thiosulfate could be further exported extracellularly via *tauE*. This inference could be further supported by the co-expression of *sqr*, *sat*, *cysH*, TST, and *tauE* within the SRBZ-1.bins.9 in JZ3_202308 (Fig. 3b). Collectively, *sqr*-containing gene clusters can provide different strategies to protect against sulfide and arsenite poisoning. Also, the ability of SQR to use sulfide as an electron donor for AnoxyP, respiration, and inorganic carbon incorporation in these *Cyanobacteriota* cannot be ignored.

### The Presence of Non-oxygen–producing PSII Containing the “Rogue” D1 Subunit in *Thermostichales*

D1 proteins encoded by *psbA* were identified in both lineages, which provide the majority of ligands to the Mn_4_CaO_5_ oxygen-evolving center (OEC) of PSII. Phylogenetic inference suggested that *Thermostichales* MAGs contain group 2 and group 4 *psbA*, while *Gloeomargaritales* MAGs only possess group 4 *psbA* (supplementary fig. S8, Supplementary Material online). Unlike the typical and dominant group 4 PsbA, the early-differentiated group 2 PsbA (also known as “rogue” D1 or rD1) has been characterized by lacking most of the OEC-binding ligands, suggesting that they may not be capable of water splitting (Cardona et al. 2015). Considering the phylogenetic placement, rD1 may act as the intermediate state which further led to the evolution of oxygen-producing PSII. It has been reported that rD1 sequences are sporadically distributed in *Cyanobacteriota*, which may have undergone frequent horizontal gene transfers (Cardona et al. 2015). This holds true for the shallow position of rD1 sequences carried by *Thermostichales* MAGs (supplementary fig. S8, Supplementary Material online). The role of the “rogue” D1-containing PSII in these MAGs might be correlated with the occurrence of oxygen-sensitive reactions, such as nitrogen fixation. This inference can be confirmed by the observed positive correlation between the expression of rD1 genes and nitrogenases (Shi et al. 2010; Zhang and Sherman 2012; Wegener et al. 2015). For Group 4 D1 proteins, sequences from *Gloeomargaritales* are clustered with eukaryotes, confirming *Gloeomargaritales* as the closest cyanobacterial lineage to eukaryotes, which is consistent with previous phylogenomic studies (Ponce-Toledo et al. 2017; Sánchez-Baracaldo et al. 2017; supplementary fig. S8, Supplementary Material online). Collectively, the existence of group 2 and 4 D1 proteins presumably allows these basal cyanobacteria to fulfill metabolic needs in different environmental conditions.

## Discussion

**Fig. 5.**
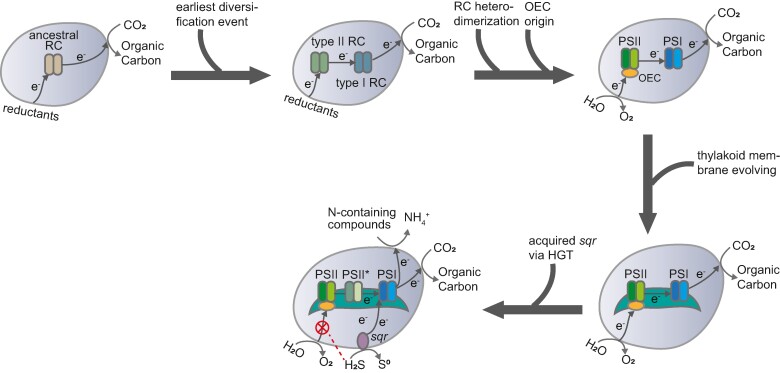
Evolutionary scenarios for the deep-branching *Cyanobacteriota*. Solid arrows indicate photosynthetic electron transfer, while dashed arrows indicate guessed electron transfer. The “forbidden” sign indicates the inhibition of sulfide on OEC of PSII. RC, reaction center; RCI, type I reaction center; RCII, type II reaction center; OEC, oxygen-evolving center; PSI, photosystem I; PSII, photosystem II; PSII*, non-oxygen–producing PSII containing group2 PsbA; *sqr*, gene encoding sulfide–quinone oxidoreductases.­

The emergence of OxyP stands as an evolutionary singularity, leading to the rise of O_2_ on early Earth and revolutionizing the planet and life on it. Photosynthetic RCs, encompassing type I and type II RCs, have been identified in at least eight bacterial phyla. However, *Cyanobacteriota* are the only group capable of OxyP, assembling both types of RC (PSI and PSII) in tandem (Hug et al. 2016; Ward et al. 2019). Other groups of bacteria evolved AnoxyP using either RCI or RCII (Hohmann-Marriott and Blankenship 2011). While the timing of the origin of OxyP and cyanobacteria remains controversial (Rasmussen et al. 2008; Schirrmeister et al. 2016), most studies generally agree that OxyP appeared prior to the Great Oxidation Event (GOE, ∼2.3 to 2.4 Ga) (Wang et al. 2018; Cardona 2019). During the time, we believe that OxyP has been progressively changed and improved from its primordially inefficient state to its current elaborate and efficient state. The most primitive RC was probably encoded by a single gene, which subsequently diverged into primordial type I and type II RCs (Sánchez-Baracaldo and Cardona 2020). Both ancestral types of RC were initially homodimeric, and later multiple gene duplication events gave rise to the evolution of heterodimeric photosystems (PSI and PSII), which are the essential structures of all known OxyP-capable organisms (Cardona and Rutherford 2019; Sánchez-Baracaldo and Cardona 2020;Fig. 5). In addition to the emergence of PSI and PSII, we suggest that the evolution of thylakoid membranes likely represented another significant evolutionary innovation in *Cyanobacteriota*. We have identified Vipp1 proteins within the *Thermostichales*, which plays a crucial role in the biogenesis of the thylakoid membrane. Our findings point to a significant role for *Thermostichales* in the evolutionary development of thylakoid membranes. Despite the absence of some photosystem auxiliary subunits in several *Thermostichales* species, their photosynthetic machinery exhibits improved integrity when compared to that of basal-branching and thylakoid-deficient *Gloeobacterales*. Consequently, we propose that the evolution of thylakoid membranes could have led to further developmental refinement of the photosystems, including an increase in auxiliary subunits. This enhancement in photosynthetic efficiency may have contributed to the ecological prominence of *Cyanobacteriota* on the early Earth. Furthermore, there are various molecular clock estimates for major cyanobacterial groups, among which the divergence time of *Thermostichales* is mostly located near the GOE, in the late Archean eon (Blank and Sánchez-Baracaldo 2010; Sánchez-Baracaldo 2015; Sánchez-Baracaldo et al. 2017) or early Paleoproterozoic era (Shih et al. 2017; Boden et al. 2021). We therefore conjecture that the evolution of thylakoid membrane may have contributed not only to the accumulation of net oxygen on early Earth but also to the onset of the GOE.

Oxygen, being the most energy-efficient electron acceptor for respiration, likely facilitated the oxidation of abundant sulfide-containing minerals on early Earth, leading to the dissolution and release of sulfur compounds into the marine environment (Lyons et al. 2009; Reinhard et al. 2009; Scott et al. 2011). We propose that these deep-branching lineages capable of OxyP may have played indispensable roles in the emergence of an oxidized world. However, sulfide has been demonstrated to inhibit typical PSII by interfering with their OEC, which in turn inhibits the occurrence of OxyP (Garcia-Pichel and Castenholz 1990; Miller and Bebout 2004). Geochemical evidence suggests that after the GOE, the global ocean tended to be sulfidic and oxygen-poor (0.1% to 10% of the present atmospheric level) for at least 1 billion years during the Proterozoic era (0.54 to 2.5 Ga; Meyer and Kump 2008; Lyons et al. 2014; Planavsky et al. 2014). Moreover, it has been proposed that the activity of anoxygenic phototrophs, such as metabolically versatile cyanobacteria, may have contributed to preventing further oxygenation of Earth's surface and maintaining the euxinic (anoxic and sulfidic) conditions of Proterozoic ocean (Johnston et al. 2009; Hamilton et al. 2016). In this context, we posit that the selective pressure stemming from the euxinic environment may have compelled *Cyanobacteriota* to evolve sulfide oxidation via the acquisition of the *sqr* gene, thereby integrating it with the photosynthetic electron transfer chain (Fig. 5). The sulfidotrophic AnoxyP currently characterized in *Cyanobacteriota* transfers electrons released during sulfide oxidation to PSI, bypassing PSII (Klatt et al. 2015). However, the trait of sulfidotrophic AnoxyP is sparsely distributed in *Cyanobacteriota* without detectable heritability (Miller and Bebout 2004; Dick et al. 2018). Additionally, the identification of the *sqr* gene on cyanobacterial plasmids located in proximity to transposable elements further supports the likelihood of horizontal transfers (Nagy et al. 2014). Although there is no explicit evidence for phylum-level HGT, our phylogenetic analyses of SQR proteins support the presence of frequent intra-phylum HGT in the *Cyanobacteriota* phylum (Fig. 4;supplementary fig. S7, Supplementary Material online). Thus, the sulfide-driven AnoxyP may not represent an innate feature in *Cyanobacteriota*. Nevertheless, we cannot rule out the possibility that an as yet unidentified deep-branching lineage may possess AnoxyP after the emergence of *Cyanobacteriota*. More importantly, for *Cyanobacteriota* living in the Proterozoic oceans, it was crucial to develop this sulfide-driven AnoxyP, which couples the consumption of toxic sulfides with energy conservation.

Furthermore, equipping both OxyP and AnoxyP can provide *Cyanobacteriota* with additional energy production advantages to fuel high-energy–consuming reactions. Specifically, experimental studies have shown that some versatile cyanobacteria perform sulfide-dependent N_2_ fixation (Belkin et al. 1982) or preferentially assimilate nitrate during AnoxyP (Klatt et al. 2015). Such coupling potentially creates new ecological adaptation opportunities for *Cyanobacteriota*, enabling the establishment of new niches and enhancing their fitness (Fig. 5). In this study, diverse strategies for assimilating nitrogen were identified in cyanobacterial MAGs, including ammonium uptake, nitrate reduction, urea utilization and atmospheric N_2_ fixation (Fig. 3;supplementary fig. S6, Supplementary Material online). Our phylogenetic results demonstrates that HGT drove the acquisition of relevant genes, including *ureABC*, *nifKDH* and *uc* (supplementary figs. S8 to S10, Supplementary Material online). It is highly probable that *Cyanobacteriota* employed either AnoxyP or OxyP, or both, to maintain a stable energy supply for nitrogen acquisition. Consequently, *Cyanobacteriota* armed with both AnoxyP and OxyP would hold a competitive advantage in sulfide-rich but nitrogen- and oxygen-depleted environments. However, experimental evidences and microbial isolates are required to verify their functional flexibility in diverse environments. This study significantly enhances our understanding of the vital yet underexplored basal lineages within *Cyanobacteriota*, offering a unique avenue for investigating the origin of *Cyanobacteriota* and their photosynthetic processes.

## Materials and Methods

### Samples Collection, Nucleic Acid Extraction, and Sequencing

A total of 22 sediment samples were collected from 8 hot springs, including DaGeJia (DG), DiReChi (DRC), GuMingQuan (GMQ), GongXiaoShe (GXS), HeHuaBianFuWa (HHBFW), ShiZiTouDuiMian (SZTDM), ShuiReBaoZha (SRBZ), and BianFu (BF), located in AngDa County of Tibet and Tengchong County of Yunnan, China. All these 22 sediment samples were subjected to DNA extraction and metagenomic sequencing. The geographical and physiochemical properties of these samples are shown in supplementary table S1, Supplementary Material online. As RNA extraction from extreme environments can be difficult, only four sediment samples successfully generated metatranscriptomes for the detection of in situ gene expression. These samples were collected from two hot springs in Tengchong County near the sampling sites for metagenomes, including three samples(JZ3_202207, JZ3_202302, JZ3_202308) from JinZe (JZ) collected in July 2022, Feb 2023, and Aug 2023, respectively, and one sample (QQ_202207) from QiaoQuan (QQ) collected in July 2022. A detailed description of the extraction of nucleic acids and sequencing methodologies can be found in supplementary Materials and Methods, Supplementary Material online.

### Metagenomic Assembly and Genome Binning

De novo metagenomic assembly and genome binning were carried out separately for each sample as described previously (Hua et al. 2019). Briefly, raw metagenomic reads for each sample were first preprocessed to obtain quality reads using custom Perl scripts (Hua et al. 2018). The quality reads for each sample were assembled independently using SPAdes v3.9.0 (Bankevich et al. 2012) with the parameters as follows: -meta -k 21,33,55,77,99,127. Each assembled data set was binned by MetaBAT v2.12.1 (Kang et al. 2015) based on tetranucleotide frequency and scaffold coverage. The coverage of scaffolds was calculated by mapping quality reads of each sample to the assembled scaffolds (length ≥ 2,500 bp) separately using BBMap v38.8 (Bushnell 2014) with the parameters “k = 15 minid = 0.97, local = t”. To improve the quality of MAGs (metagenome-assembled genomes), RefineM v0.1.1 (Parks et al. 2017) was used to identify and remove outlier scaffolds with divergent GC content, coverage, or tetranucleotide signatures. For all MAGs, genome completeness, contamination, and strain heterogeneity were evaluated using CheckM v1.0.12 (Parks et al. 2015). The generated MAGs were assigned taxonomic classification through GTDB-Tk v2.2.5 (Chaumeil et al. 2022). Finally, a total of 36 MAGs belonging to *Thermostichales* and *Gloeomargaritales* were selected for further analysis.

### Metatranscriptomic Analysis

Raw metatranscriptomic reads were filtered using Sickle v1.33 (https://github.com/najoshi/sickle) with the parameters “-t sanger –quiet -l 50”. Considering some MAGs in this study were redundant, 9 out of 36 MAGs were manually selected as representative genomes based on 95% ANI and high genome quality before performing transcript-level analysis. Specifically, genomes with higher completeness, fewer scaffolds (or a larger N50 value) and a greater number of genes relevant to photosynthetic system were considered high quality and chosen as representatives. It should be additionally noted that two representative genomes (DG_2_bins_126 and DG02_1_bins_92) were retained in species_2 of *Gloeomargaritales* due to the fact that the ANI value between DG_2_bins_126 and the other genomes of species_2 being around 95% (supplementary table S3, Supplementary Material online) and its position on a separate branch within the phylogenomic tree (Fig. 1). Subsequently, high-quality metatranscriptomic reads were mapped to these non-redundant representative genomes using coverM in contig mode with settings of “-m rpkm -p bwa-mem –min-read-percent-identity 0.95 –min-read-aligned-percent 0.5” (https://github.com/wwood/CoverM), and the expression level for each gene was normalized to reads per kilobase per million mapped reads (RPKM).

### Functional Annotation of MAGs

For these MAGs, gene prediction was performed using Prodigal v2.6.3 (Hyatt et al. 2010) with the “-p single -m” option and 16S rRNA genes were detected using RNAmmer (Lagesen et al. 2007). For functional annotation, proteins were queried against databases including NCBI-nr, KEGG, and eggNOG using DIAMOND v0.7.9 with a cutoff *E* < 1e−5. Proteins were also annotated by KofamScan v1.3.0 (Aramaki et al. 2020) using a customized HMM database of KEGG Orthologs (KOfam v99.0), which assigns K numbers via HMMER/HMMSEARCH. Additionally, proteins were analyzed with Interproscan v5.54-87.0 (Jones et al. 2014) to identify protein domain information (PFAMs, TIGRFAMs, and IPR domains). The ANI between all MAGs was calculated using the OrthoANI-Usearch (Yoon et al. 2017) (OAU) software from EZbiocloud. The AAI shared by any two MAGs was calculated using CompareM v0.0.17 (https://github.com/dparks1134/CompareM) with the “aai_wf” option.

### Phylogenomic and Phylogenetic Analyses

#### Phylogenomics

Two different marker sets, including a concatenation of 31 conserved proteins (Shih et al. 2013) and 120 bacterial marker genes in the GTDB-TK database, were retrieved from 36 MAGs and 165 selected public cyanobacterial reference genomes to construct the phylogenomic tree. AMPHORA2 (Wu and Scott 2012) was used to identify orthologues of 31 marker proteins. Individual marker protein were aligned using MAFFT v7.505 (Katoh and Standley 2013) with the parameters “–maxiterate 1000 –localpair”, and poorly aligned positions were trimmed by TrimAL v1.4.rev22 (Capella-Gutiérrez et al. 2009) with the parameters “-gt 0.9 -cons 60”. Individual protein alignments were then concatenated resulting in an alignment of 8307 sites. Genomes were processed using GTDB-Tk v2.2.5 to identify 120 bacterial marker proteins and a concatenated multiple sequence alignment (MSA) was created. Two maximum-likelihood trees were constructed using IQ-TREE v1.6.10 (Nguyen et al. 2015) with the parameters “-alrt 1000 -bb 1000”, which chose LG + R10 as the best-fit models according to Bayesian Information Criterion (BIC).

#### Vipp1/PspA Family Proteins

Sequences assigned to K03969 encoding Vipp1/PspA family proteins were downloaded from the KEGG GENES database. Of these, 257 sequences were selected as reference sequences for the phylogenetic tree, including eukaryotic, archaeal, and bacterial Vipp1/PspA proteins to cover a broad distribution of homologs across the tree of life. In addition to KO assignment, Vipp1/PspA homologs were collected from 36 MAGs by performing BLASTP search using reference sequences as a query, and then filtered based on length (>200 amino acids) and bit score (>250). In total, 298 Vipp1/PspA sequences were selected, aligned using MUSCLE v3.8.31 (Edgar 2004) with default parameters, and subsequently trimmed with TrimAL v1.4.rev22 using the parameters “-gt 0.9 -cons 60”. The unique transit sequence (a N-terminal extension of 1 to 72 amino acids) in the plant Vipp1 protein was removed and realigned using MUSCLE v3.8.31 with default parameters. Maximum-likelihood phylogeny was inferred with IQ-TREE v1.6.10 with the parameters “-alrt 1000 -bb 1000” using the LG + F + R9 model, which was the best-fit mode according to the BIC (see supplementary Materials and Methods, Supplementary Material online, for further details of Vipp1/PspA protein structure analysis).

#### Sulfide–Quinone Reductase

Based on functional annotation of MAGs (see supplementary Materials and Methods, Supplementary Material online, for a detailed description), 43 protein sequences assigned to COG01850 (sulfide–quinone reductase, SQR) were extracted from the MAGs of two lineages. Then, we searched for homologs of these sequences in the 65,703 representative genomes from the GTDB r207 using Diamond blastp v2.1.7.161 with the parameters “–max-target-seqs 25 –evalue 0.00001”. Moreover, 49 SQR sequences (types I–VI) identified from previous study were also added to the reference dataset (Marcia et al. 2010). A total of 194 SQR sequences were aligned using MUSCLE v3.8.31 with default parameters and trimmed by TrimALv1.4.rev22 with the parameters “-gt 0.9 -cons 60”. A maximum-likelihood phylogenetic tree was constructed using IQ-TREE v1.6.10 with the parameters “-alrt 1000 -bb 1000”. The best-fit model WAG + R8 was chosen based on the BIC score. Gene cluster organization of *sqr* was generated with the R package gggenes (https://cran.r-project.org/web/packages/gggenes/index.html). For the generation of a comprehensive phylogenetic tree, homologs of SQR were retrieved from IMG genomic databases (Markowitz et al. 2012) by KO-based searching (K17218 and K22470), followed by manual inspection. In total, 779 SQR proteins (including 731 bacterial, 44 archaeal, and 12 eukaryotic sequences) were selected, aligned using MAFFT v7.505 with the l-INS-I mode, and poorly aligned regions were removed using TrimAL v1.4.rev22 with the parameters “-gt 0.9 -cons 60”. The phylogeny was inferred in IQ-TREE v1.6.10 with the parameters “-alrt 1000 -bb 1000 -safe” under the LG + R10 model, which was the best-fit model according to the BIC.

For other functional genes, including *psbA* and nitrogen metabolism–related genes (*ureABC*, *nifKDH*, and *uc*), see supplementary Materials and Methods, Supplementary Material online, for a detailed description of the phylogenetic analysis. All trees were visualized and modified using iTOL (Letunic and Bork 2021).

## Supplementary Material

msae151_Supplementary_Data

## Data Availability

All genomes in our study were submitted to the NCBI database. The BioProject number is PRJNA998252. The genome accession numbers are JAUQUV000000000-JAUQUZ000000000, JAUQVA000000000-JAUQVZ000000000, and JAUQWA000000000-JAUQWE000000000. All data needed to evaluate the conclusions in the paper are present in the main text and the Supplementary information.
