# Supplementary material for: Exploring the Origins and Evolution of Oxygenic and Anoxygenic Photosynthesis in Deeply Branched Cyanobacteriota
Source: Mol Biol Evol. 2024 Jul 23;41(8):msae151. doi: 10.1093/molbev/msae151 (PMC11304991; doi:10.1093/molbev/msae151)
Supplement: msae151_Supplementary_Data [file msae151_supplementary_data.zip › Tan et al_Supplementary_text_figures_final_submit.pdf]

Exploring the origins and evolution of oxygenic and anoxygenic photosynthesis in deeply branched *Cyanobacteriota*

Sha Tan, Lan Liu, Jian-Yu Jiao, Meng-Meng Li, Chao-Jian Hu, Ai-Ping Lv, Yan-Ling Qi, Yu-Xian Li, Yang-Zhi Rao, Yan-Ni Qu, Hong-Chen Jiang, Rochelle M. Soo, Paul N. Evans, Zheng-Shuang Hua*, Wen-Jun Li*

*Corresponding author: Zheng-Shuang Hua and Wen-Jun Li

Email: hzhengsh@ustc.edu.cn; liwenjun3@mail.sysu.edu.cn

This PDF file includes:

Materials and Methods	Page 2-4
Figures S1 to S11	Page 5-16
Legends for Tables S1 to S8	Page 17
References	Page 18

Other supplementary materials for this manuscript include the following:

Tables S1 to S8

Materials and Methods

Nucleic acids extraction and sequencing

All the sediment samples were collected into 50 ml falcon tubes with sterile spatulas and spoons and stored in liquid nitrogen before being transported to the laboratory. The DNA extraction and metagenomic sequencing were processed as described previously (Hua et al. 2018). Briefly, community DNA was extracted from approximately 20 g of sediment material using PowerSoil DNA Isolation kit (MoBio). The concentrations of DNA were measured by Qubit fluorometer. Libraries with an insert size of 350 bp were constructed using the M220 Focused-ultrasonicator NEBNext and Ultra II DNA library prep kit. Libraries of twenty-two samples were sequenced using Illumina Hiseq 4000 instruments at Beijing Novogene Bioinformatics Technology Co., Ltd (Beijing, China). The amount of raw metagenomic sequence data for each sample was approximately 30 giga base pairs (Gbp).

Total RNA of each sample was extracted using RNeasy PowerSoil Total RNA kit (QIAGEN), with the metatranscriptomic sequence data generated on an Illumina HiSeq 4000 platform (paired-end 150-bp mode) at the Guangdong Magigene Biotechnology Co., Ltd. (Guangzhou, China). 20-30 Gbp of raw metatranscriptomic sequence data for each sample were generated.

Phylogenetic analyses

PsbA. Protein sequences with K02703 encoding photosystem II PsbA (D1) protein were extracted from MAGs and filtered based on a length cut-off of 300 amino acids. Then, 31 PsbA protein sequences were clustered with 97% identity by CD-HIT v4.8.1 (Fu et al. 2012), resulting in 8 representative sequences being kept. Combined with the selected reference sequences from a previous study (Cardona et al. 2015), a total of 93 PsbA sequences were aligned using Clustal Omega (Sievers and Higgins 2014) with the parameters "--max-guidetree-iterations=10000 --max-hmm-iterations=10000000". Maximum likelihood analysis of the final phylogeny using PhyML v3.1 (Guindon et al. 2010) with the parameters "-d aa -b -2 -f e -v e -c 4 -a e -s NNI -m LG -o l". Branch support was calculated with the approximate likelihood ratio test option.

Urea carboxylase. Urea carboxylase (UC) sequences were identified in the MAGs based on functional annotation (K01941). Reference datasets were retrieved from the IMG genomic databases (Markowitz et al. 2012) by searching for enzymes with EC 6.3.4.6 numbers. All reference sequences were clustered with 90% identity by CD-HIT v4.8.1. These sequences were then grouped by source organism genus, and only one representative sequence was retained in each genus, resulting in 646 representative sequences being kept. All UC sequences were aligned using MUSCLE v3.8.31 with the default parameters, and trimmed by TrimAL v1.4.rev22 with the parameters "-gt 0.9 -cons 60". The maximum-likelihood phylogeny was reconstructed by IQ-TREE v1.6.10 (using the LG+F+R10 model chosen based on the BIC score) with the parameters "-alrt 1000 -bb 1000".

Nitrogenase (NifHDK). According to the functional annotation, the protein sequences for NifHDK were extracted from the MAGs which encoded all three genes. Reference sequences for NifHDK were downloaded from IMG genomic databases through KO-based searching (K02588, K02586, and K02591 for NifH, NifD, and NifK). Reference genomes that did not contain the complete NifHDK sequences were removed by manual filtering, and only one representative genome with these sequences was kept from each genus for further analyses. By separating the reference genomes by genus, 636 microbial representative genomes were obtained. All NifHDK sequences were then individually aligned with MAFFT-LINSi v7.505 and trimmed using TrimAl v1.4.rev22 with the "gappyout" option. A maximum-likelihood tree was constructed on a concatenated alignment of NifHDK by IQ-TREE v1.6.10 with the parameters "-m MFP -alrt 1000 -bb 1000 –bnni ".

Urease (UreABC). Proteins for UreA, UreB, and UreC were identified in the MAGs based on functional annotation. Reference sequences were download from the IMG genomic databases by enzyme ID-based search (EC 3.5.1.5). Manual filtering was performed to retain genomes containing the UreABC sequences, with each subunit then independently aligned using MUSCLE v3.8.31 with the default parameters and trimmed using TrimAl v1.4.rev22 with the "-gt 0.95 -cons 50" option, and then concatenated. In

order to simplify the phylogenetic tree, the concatenated UreABC gene sequences were clustered using CD-HIT v4.8.1 with the parameters "-aS 1 -c 0.8 -g 1". A total of 757 representative UreABC sequences were retained for phylogenetic reconstruction. The maximum-likelihood phylogeny was reconstructed by IQ-TREE v1.6.10 with the parameters "-alrt 1000 -bb 1000", in which "LG+F+R10" was determined as the best model. Additionally, UreABC sequences from cyanobacterial genomes were selected to construct the *Cyanobacteriota*-specific tree. Phylogenetic trees were constructed using the same procedures as above with "LG+R4" as the best model. All trees were visualized and modified using iTOL.

Vipp1 and PspA proteins sequnece and structure analysis

The multiple sequence alignment between selected representative Vipp1/PspA proteins was constructed with Clustal Omega by JalView v2.11.2.4 (Waterhouse et al. 2009) and colored by the percentage of identity. Secondary structure predictions were performed using PSIPRED v4.0 (Buchan and Jones 2019). The 3D structures of Vipp1 and PspA proteins were predicted using SWISS-MODEL (Waterhouse et al. 2018) and TrRosetta (Du et al. 2021). PyMOL (Lilkova et al. 2015) software was used to further visualize and manipulate 3D structures.

Figures S1 to S11

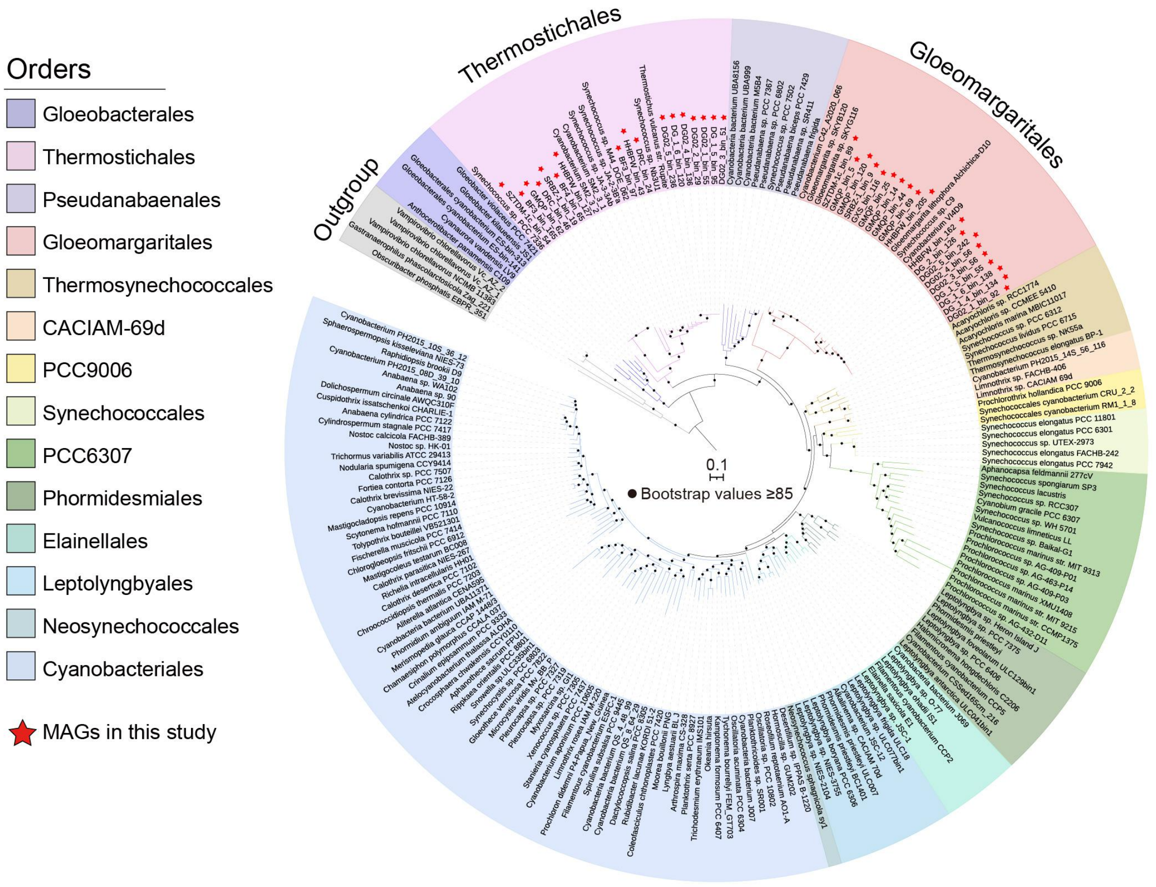

Fig. S1. The phylogenetic tree was generated based on the concatenated 120 bacterial marker proteins. References from the class *Vampirovibrionia* were treated as outgroups. Branches and labels are colored according to the GTDB r214 order-level classification. Nodes with bootstrap value ⩾85 are indicated as solid circle. 36 MAGs generated in this study are marked by red stars.

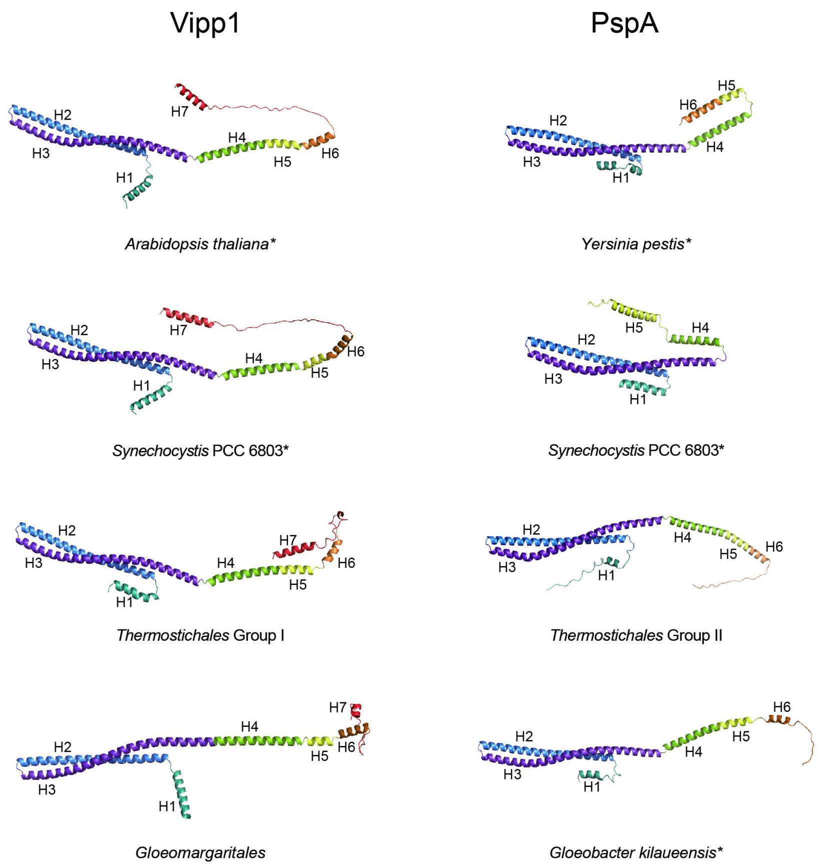

Fig. S2. The tertiary structures of selected Vipp1 and PspA proteins. α-helices are marked by different colors and labeled H1-H7.

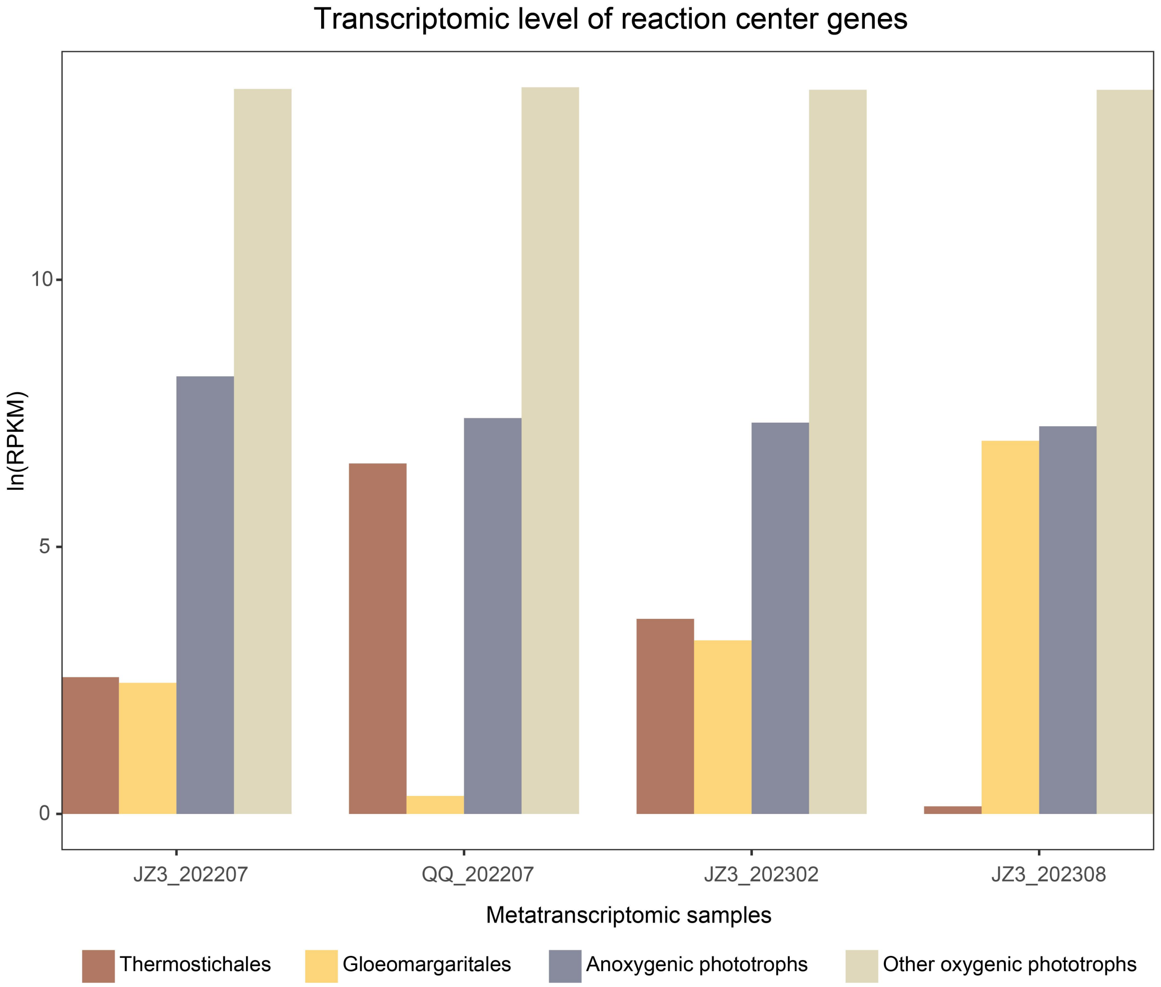

Fig. S3. *In-situ* activities of genes associated with photosynthetic reaction centers from different photosynthetic groups in four metatranscriptomes.

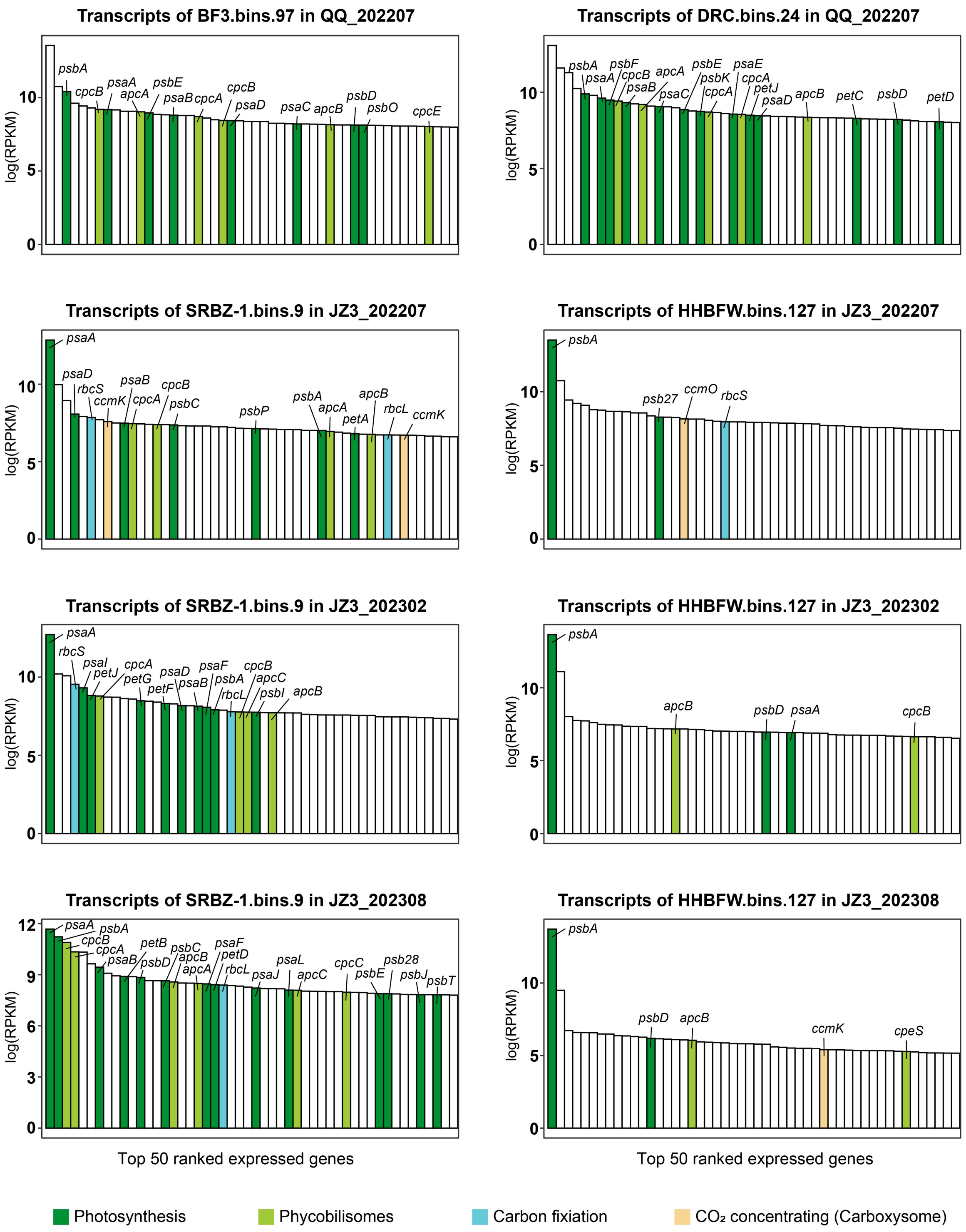

Fig. S4. Expressed gene of representative MAGs (BF3.bins.97, DRC.bins.24, HHBFW.bins.127 from *Thermostichales* and SRBZ-1.bins.9 from *Gloeomargaritales*) in four metatranscriptomes.

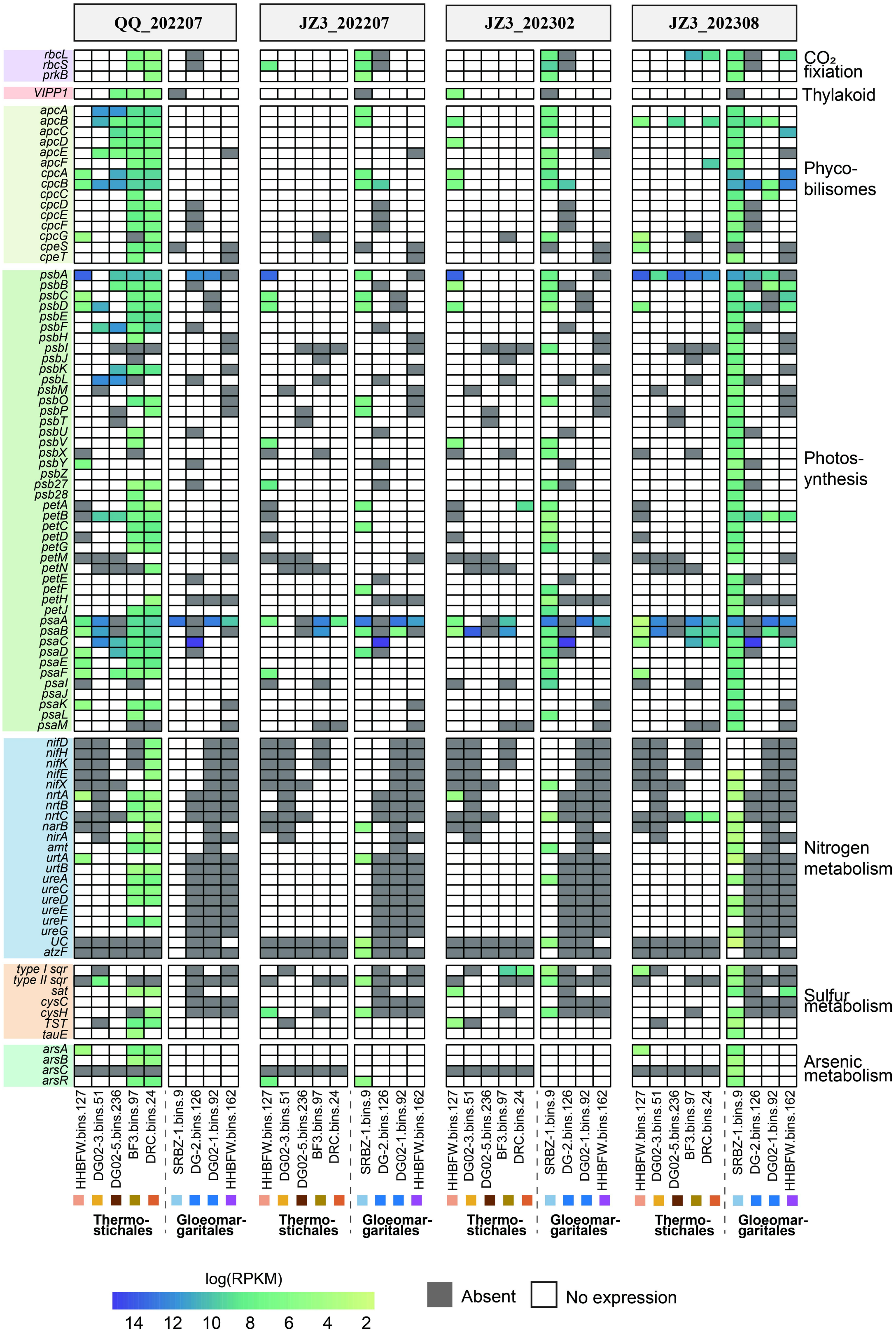

Fig. S5. A detailed demonstration of the *in-situ* activity of each gene in Fig. 3(**B**).

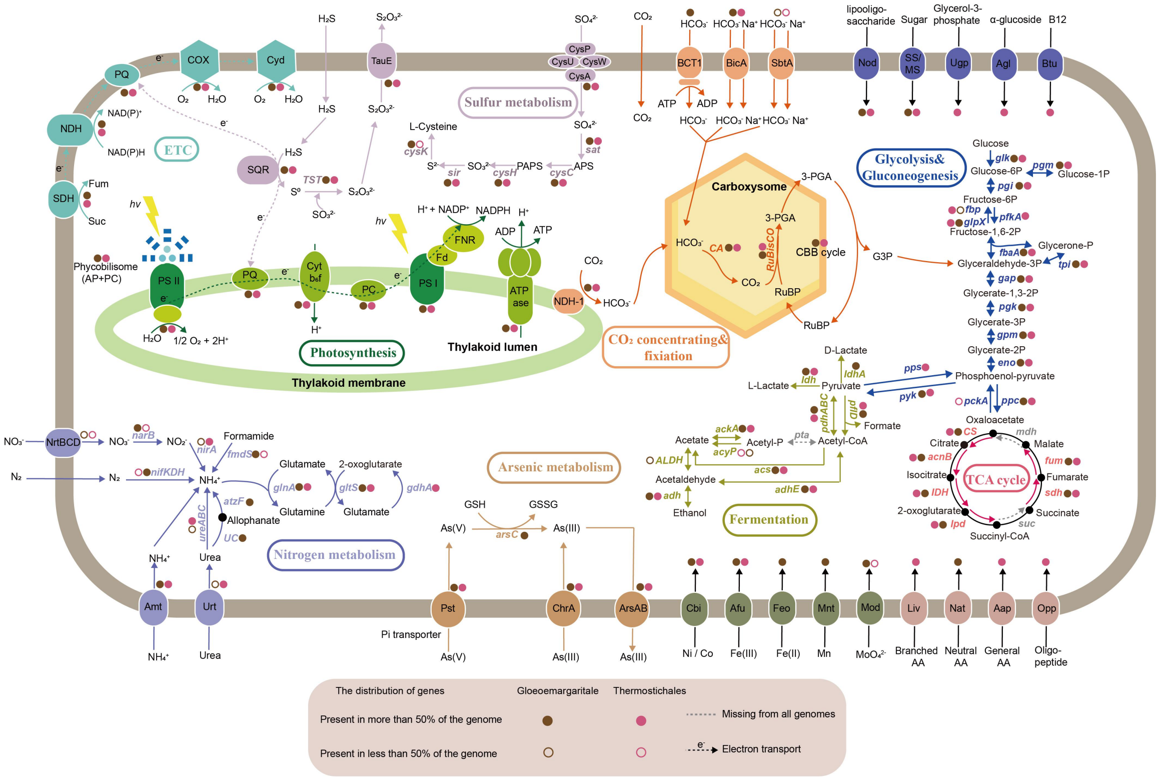

Fig. S6. Metabolic potential of *Thermostichales* and *Gloeoemargaritale* MAGs. Pathways and genes associated with photosynthesis, carbon, nitrogen, sulfur, arsenic, fermentation, energy conservation, and various transporters are shown. Details about the genes are provided in Dataset S4.

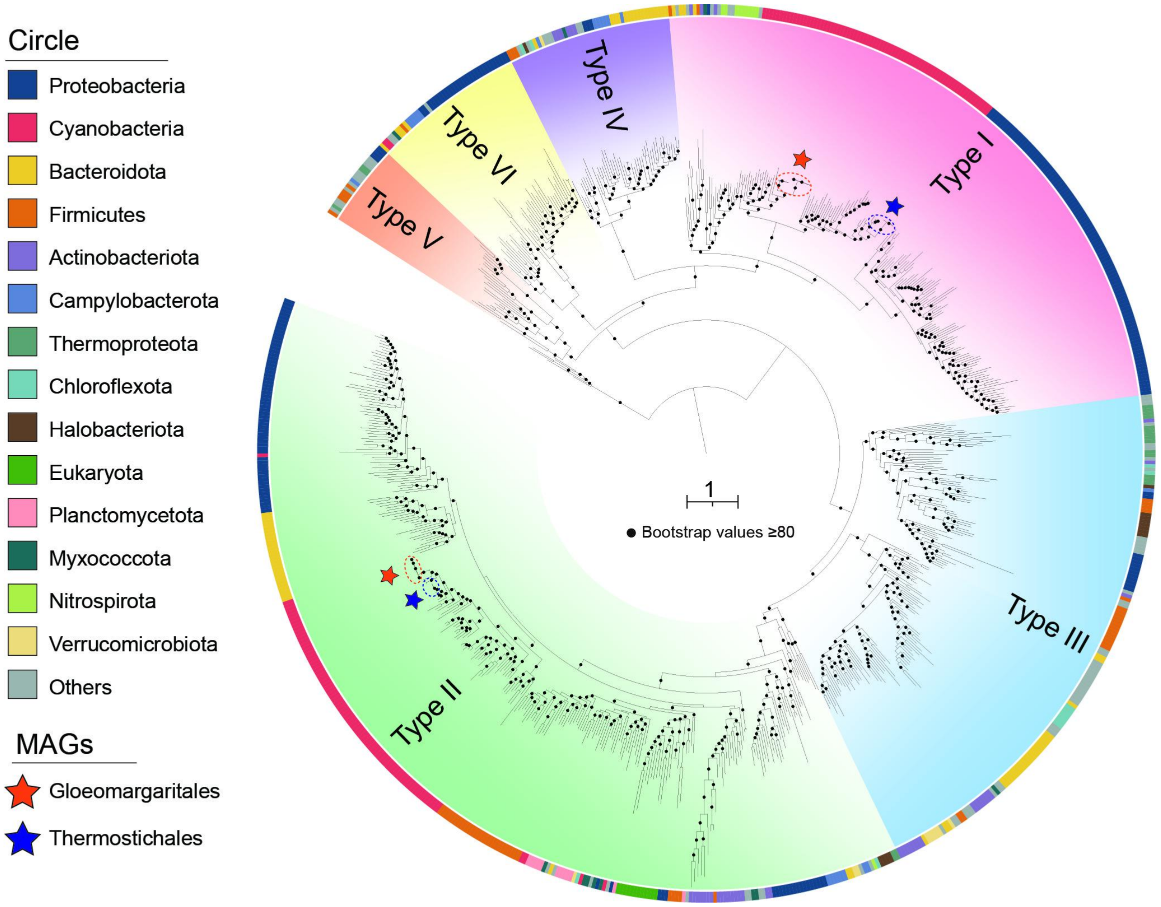

Fig. S7. A phylogenetic analysis of SQR homologs across bacteria, archaea and eukaryote. The maximum likelihood tree includes 779 sequences and is rooted by 6 flavocytochrome c:sulfide dehydrogenase (FCSD) sequences. SQR homologs from Type I to Type VI are colored within the corresponding branches in the tree. Circle from the outermost indicates the taxonomic affiliation of the genes at the phylum level. Nodes with bootstrap values ⩾ 80 were marked by black dots. Sequences from *Gloeomargaritales* and *Thermostichales* MAGs are marked by orange and blue stars.

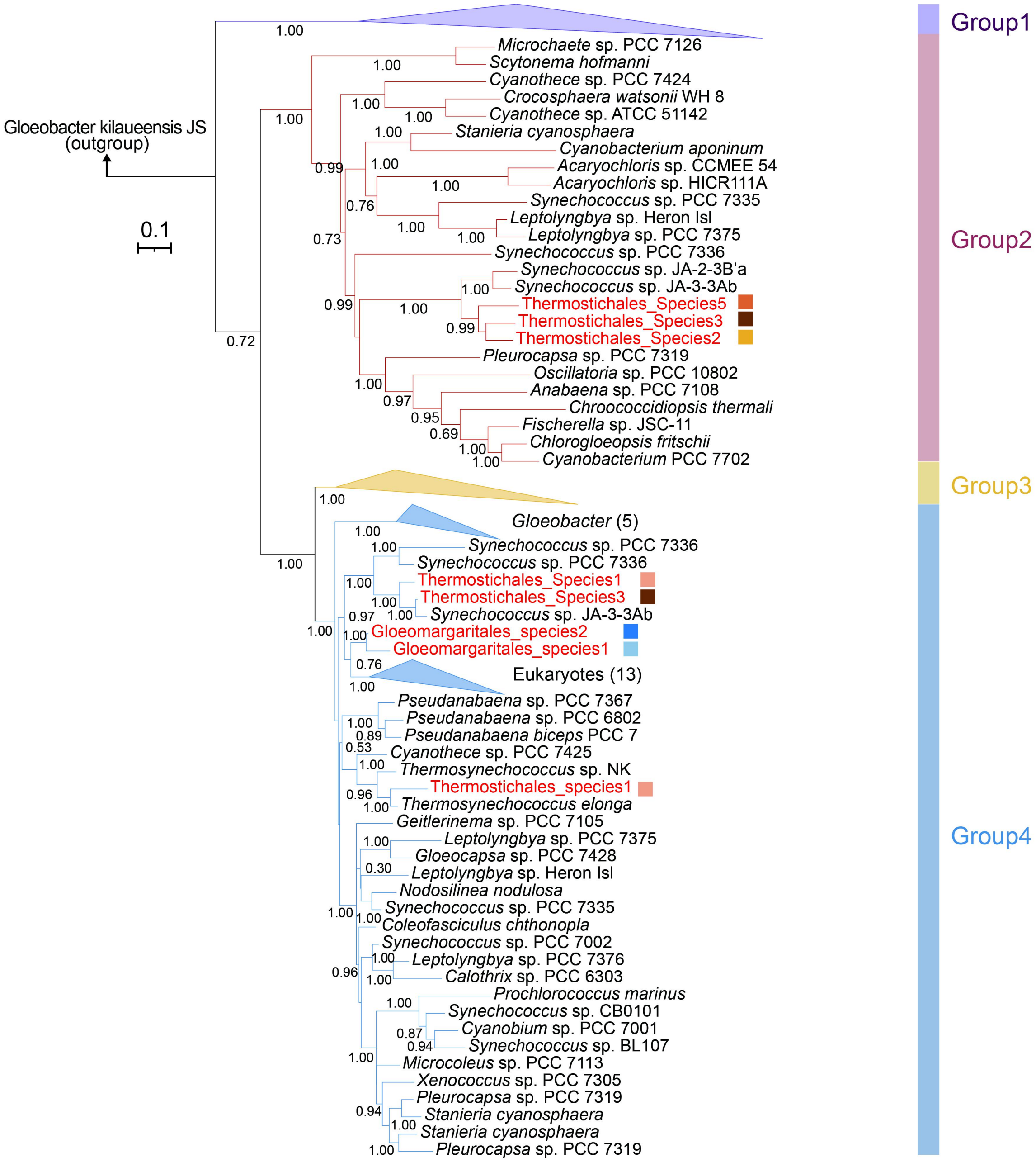

Fig. S8. Maximum likelihood phylogenetic tree of PsbA (D1 proteins). The outgroup is a PsbA sequence from *Gloeobacter kilaueensis* JS. Branches are colored by Group 1–4 sequences. Branch supports are expressed as aLRT probabilities. PsbA sequences from the MAGs in this study are marked in red and taxonomic information is represented by colored squares on the right side, following the same color scheme as depicted in Fig. 1.

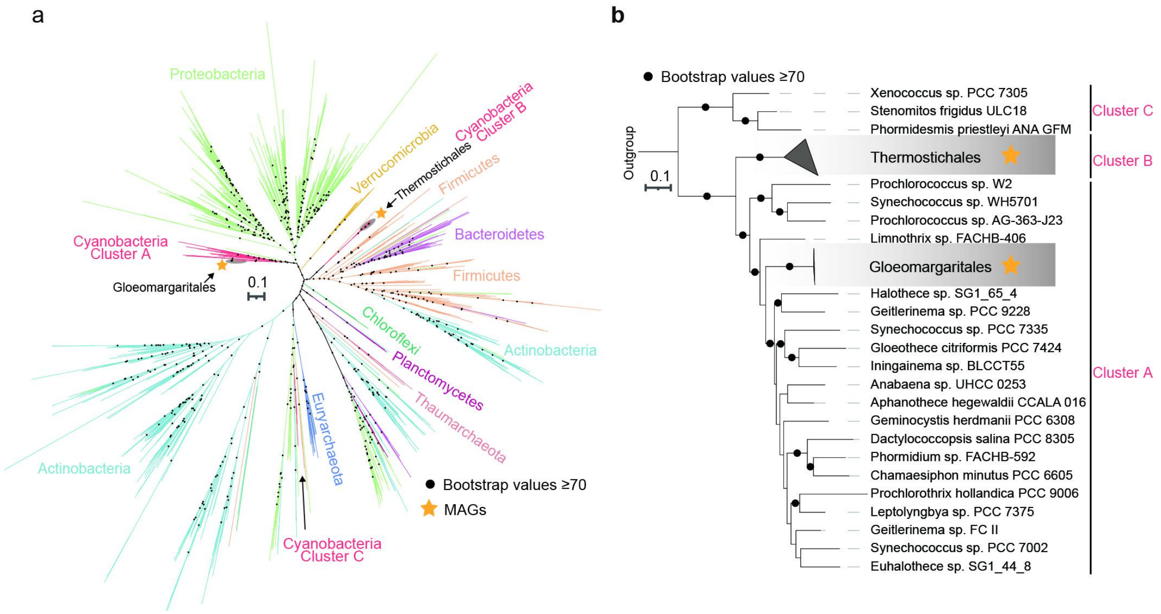

Fig. S9. Phylogenetic tree of concatenated UreABC extracted from (a) publicly prokaryotic reference genomes and (b) cyanobacterial genomes. Both trees were calculated with 1000 bootstrap, nodes with bootstrap value ⩾ 70 are indicated as solid circle. Genes encoded in *Gloeomargaritales* and *Thermostichales* MAGs are circled by gray and marked by yellow stars.

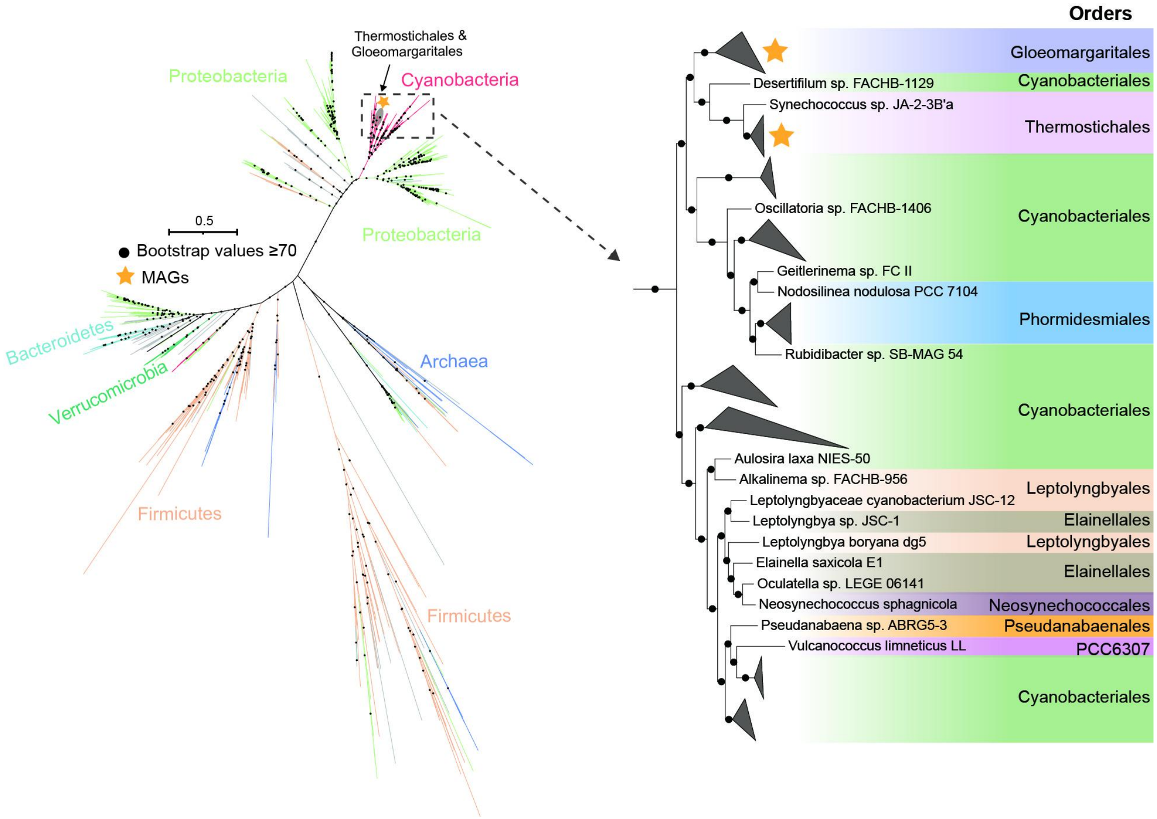

Fig. S10. Phylogenetic tree of concatenated NifHKD. Branches of unroot tree are colored by phylum. Branches of zoomed-in cyanobacterial clade are colored by order. Nodes with bootstrap value ⩾70 are indicated as solid circle. Genes encoded in *Gloeomargaritales* and *Thermostichales* MAGs are circled by gray and marked by yellow stars.

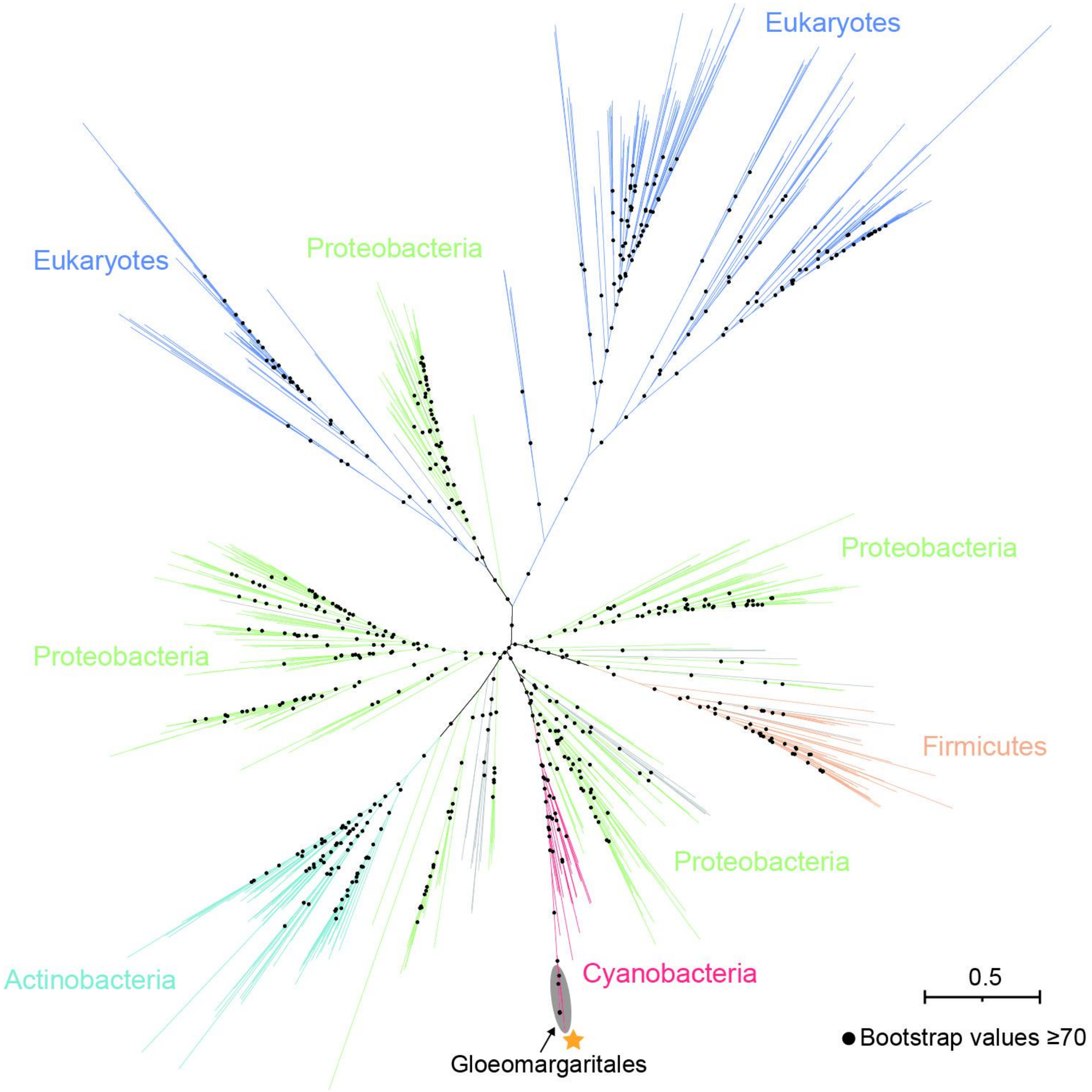

Fig. S11. Phylogenetic tree of urea carboxylase (UC). Reference sequences include the urea carboxylase domains of the urea amidolyase proteins and the urea carboxylase proteins. Branches are colored by phylum. Nodes with bootstrap value ⩾70 are indicated as solid circle. Genes form *Gloeomargaritales* MAGs are circled by gray and marked by yellow stars.

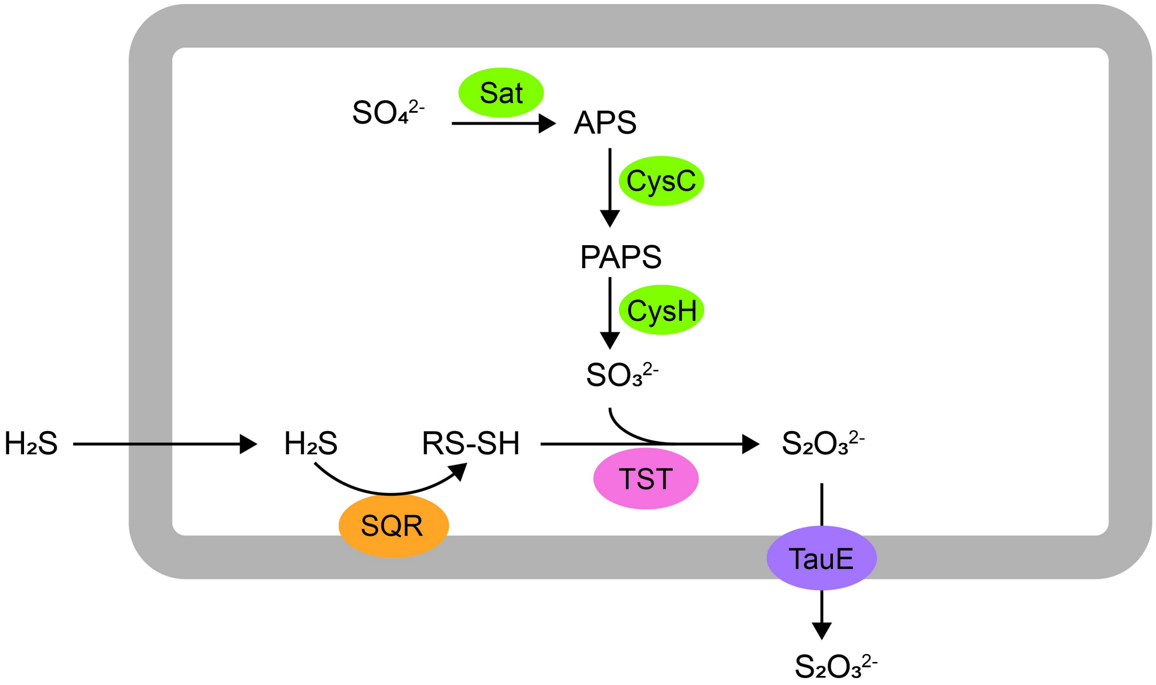

Fig. S12. Proposed alternative model of sulfide oxidation. Scheme showing enzymes involved in the possible sulfide oxidation pathway. Abbreviations: SQR, sulfide:quinone oxidoreductase; Sat, sulfate adenylyltransferase; CysC, adenylylsulfate kinase; CysH, phosphoadenosine phosphosulfate reductase; TST, thiosulfate sulfotransferase; TauE, sulfite and/or thiosulfate exporter; APS, 5'-Adenylyl sulfate; PAPS, 3'-Phospho-5'-adenylyl sulfate.

Legends for Table S1 to S8

Table S1 (separate file). The geographical and physiochemical information of twenty-two sediment samples.

Table S2 (separate file). Genome statistics of the 36 MAGs recovered from the metagenome assembly.

Table S3 (separate file). OrthoANI matrix of the MAGs of *Thermostichales* and *Gloeomargaritales* and reference species.

Table S4 (separate file). AAI matrix of the MAGs of *Thermostichales* and *Gloeomargaritales* and reference species.

Table S5 (separate file). The 16S rRNA gene sequence identity matrix of the MAGs of *Thermostichales* and *Gloeomargaritales* and reference species.

Table S6 (separate file). Distribution of gene involved in thylakoid membrane lipid biosynthesis in MAGs and reference genomes.

Table S7 (separate file). Metabolic potential of MAGs and reference genomes.

Table S8 (separate file). Gene expression of representative *Thermostichales* and *Gloeomargaritales* MAGs in four hot spring metatranscriptomes.

References

Buchan DWA, Jones DT. 2019. The PSIPRED protein analysis workbench: 20 years on. *Nucleic. Acids Res.* 47:W402–W407.

Cardona T, Murray JW, Rutherford AW. 2015. Origin and evolution of water oxidation before the last common ancestor of the Cyanobacteria. *Mol. Biol. Evol.* 32:1310–1328.

Du Z, Su H, Wang W, Ye L, Wei H, Peng Z, Anishchenko I, Baker D, Yang J. 2021. The trRosetta server for fast and accurate protein structure prediction. *Nat. Protoc.* 16:5634–5651.

Fu L, Niu B, Zhu Z, Wu S, Li W. 2012. CD-HIT: accelerated for clustering the next-generation sequencing data. *Bioinformatics* 28:3150–3152.

Guindon S, Dufayard J-F, Lefort V, Anisimova M, Hordijk W, Gascuel O. 2010. New algorithms and methods to estimate maximum-likelihood phylogenies: assessing the performance of PhyML 3.0. *Syst. Biol.* 59:307–321.

Hua ZS, Qu Y-N, Zhu Q, Zhou E-M, Qi Y-L, Yin Y-R, Rao Y-Z, Tian Y, Li Y-X, Liu L, et al. 2018. Genomic inference of the metabolism and evolution of the archaeal phylum Aigarchaeota. *Nat. Commun.* 9:2832.

Lilkova E, Petkov P, Ilieva N, Litov L. 2015. The PyMOL molecular graphics system, version 2.0. *Schrodinger, LLC.*

Markowitz VM, Chen I-MA, Palaniappan K, Chu K, Szeto E, Grechkin Y, Ratner A, Jacob B, Huang J, Williams P, et al. 2012. IMG: the Integrated Microbial Genomes database and comparative analysis system. *Nucleic. Acids Res.* 40:D115-122.

Sievers F, Higgins DG. 2014. Clustal Omega, accurate alignment of very large numbers of sequences. *Methods Mol. Biol.* 1079:105–116.

Waterhouse A, Bertoni M, Bienert S, Studer G, Tauriello G, Gumienny R, Heer FT, de Beer TAP, Rempfer C, Bordoli L, et al. 2018. SWISS-MODEL: homology modelling of protein structures and complexes. *Nucleic Acids Res* 46:W296–W303.

Waterhouse AM, Procter JB, Martin DMA, Clamp M, Barton GJ. 2009. Jalview Version 2--a multiple sequence alignment editor and analysis workbench. *Bioinformatics* 25:1189–1191.
